# Dynamic expression of SNAI2 in prostate cancer predicts tumor progression and drug sensitivity

**DOI:** 10.1002/1878-0261.13140

**Published:** 2022-02-11

**Authors:** Ying Z. Mazzu, YuRou Liao, Subhiksha Nandakumar, Martin Sjöström, Lina E. Jehane, Romina Ghale, Barani Govindarajan, Travis A. Gerke, Gwo‐Shu Mary Lee, Jian‐Hua Luo, Sreenivasa R. Chinni, Lorelei A. Mucci, Felix Y. Feng, Philip W. Kantoff

**Affiliations:** ^1^ Department of Medicine Memorial Sloan Kettering Cancer Center New York NY USA; ^2^ Center for Molecular Oncology Memorial Sloan Kettering Cancer Center New York NY USA; ^3^ Department of Radiation Oncology University of California San Francisco CA USA; ^4^ Division of Hematology and Oncology Department of Medicine University of California San Francisco CA USA; ^5^ Department of Pathology Wayne State University Detroit MI USA; ^6^ Prostate Cancer Clinical Trials Consortium New York NY USA; ^7^ Department of Medicine Dana‐Farber Cancer Institute Boston MA USA; ^8^ Department of Pathology University of Pittsburgh School of Medicine PA USA; ^9^ Department of Epidemiology Harvard T.H. Chan School of Public Health Boston MA USA; ^10^ Helen Diller Family Comprehensive Cancer Center University of California San Francisco CA USA; ^11^ Department of Urology University of California San Francisco CA USA

**Keywords:** dasatinib, DNA methylation, HDAC inhibitor, prostate cancer, SNAI2, TMPRSS2‐ERG

## Abstract

Prostate cancer is a highly heterogeneous disease, understanding the crosstalk between complex genomic and epigenomic alterations will aid in developing targeted therapeutics. We demonstrate that, even though snail family transcriptional repressor 2 (*SNAI2*) is frequently amplified in prostate cancer, it is epigenetically silenced in this disease, with dynamic changes in *SNAI2* levels showing distinct clinical relevance. Integrative clinical data from 18 prostate cancer cohorts and experimental evidence showed that gene fusion between transmembrane serine protease 2 (*TMPRSS2*) and ETS transcription factor ERG (*ERG*) (*TMPRSS2–ERG* fusion) is involved in the silencing of *SNAI2*. We created a silencer score to evaluate epigenetic repression of *SNAI2*, which can be reversed by treatment with DNA methyltransferase inhibitors and histone deacetylase inhibitors. Silencing of *SNAI2* facilitated tumor cell proliferation and luminal differentiation. Furthermore, *SNAI2* has a major influence on the tumor microenvironment by reactivating tumor stroma and creating an immunosuppressive microenvironment in prostate cancer. Importantly, *SNAI2* expression levels in part determine sensitivity to the cancer drugs dasatinib and panobinostat. For the first time, we defined the distinct clinical relevance of *SNAI2* expression at different disease stages. We elucidated how epigenetic silencing of *SNAI2* controls the dynamic changes of *SNAI2* expression that are essential for tumor initiation and progression and discovered that restoring *SNAI2* expression by treatment with panobinostat enhances dasatinib sensitivity, indicating a new therapeutic strategy for prostate cancer.

## Graphical abstract

Here, the authors define, for the first time, the distinct clinical relevance of *SNAI2* expression at different disease stages of prostate cancer (PC). They elucidated how the epigenetic silencing of *SNAI2* controls the dynamic changes in *SNAI2* expression that are essential for tumor initiation and progression. Importantly, restoring *SNAI2* expression by LBH589 (HDACi) enhances dasatinib sensitivity, indicating a new therapeutic strategy for PC.

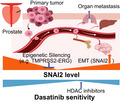

**Abbreviations**
5‐Aza‐dC: 5‐Aza‐2′‐deoxycytidine
ANXA1: annexin A1
AR: androgen receptor
CAV1: caveolin 1
CAVIN1: caveolae‐associated protein 1
CNA: copy number alteration
DFS: disease‐free survival
DNMT: DNA methyltransferase
DNMTi: DNA methyltransferase inhibitor
EMT: epithelial–mesenchymal–transition
EPHA2: EPH receptor A2
FGA: fraction of genome alteration
FGFR2: fibroblast growth factor receptor 2
GSEA: gene set enrichment analysis
HDAC: histone deacetylase
HDACi: histone deacetylase inhibitor
MSMB: microseminoprotein beta
NCOA2: nuclear receptor coactivator 2
OS: overall survival
PC: prostate cancer
PFS: progression‐free survival
SNAI2: snail family transcriptional repressor 2
T2E: TMPRSS2‐ERG
TCGA: The Cancer Genome Atlas
VIM: vimentin

## 1 Introduction

Comprehensively integrated ‘omics’ data provide insights into the molecular‐genetic heterogeneity of prostate cancer (PC), which contributes to diagnostic, prognostic, and therapeutic decision‐making [1, 2, 3]. Genomic alterations in PC include somatic mutations, gene deletions or amplifications, and chromosomal rearrangements, which contribute to different disease stages. ETS‐positive fusion (e.g., *TMPRSS2‐ERG*, *TMPRSS2‐ETV1*) occurs in more than 60% of primary PCs. SPOP mutation, the most common recurrent point mutation in PC [4], which is mutually exclusive of the ETS fusion‐positive subclass [1], occurs in 6**–**15% of primary PCs. The deletion or mutation of key tumor suppressors (e.g., PTEN, p53, CHD1) have been identified as the drivers of metastatic progression of PC [1, 5]. Androgen receptor (AR) gene amplification, mutation, and splice variants occur in 60% of castration‐resistant PCs, while DNA repair gene deficiency is a major contributor to progression in castration‐resistant disease [3, 6, 7]. In a similar manner to genomic alteration, epigenomic regulation contributes to the complex heterogeneity of PC at different stages of disease. DNA hypermethylation participates in cancer initiation and progression by regulating genes associated with DNA repair, the cell cycle, apoptosis, and cell adhesion [8]. Conversely, DNA hypomethylation is more frequently detected in metastatic than early‐stage PC [9, 10].

The crosstalk between genomic and epigenomic alterations could collaboratively establish aberrant precursor cell populations and differentiation lineage in PC. Epigenetic aberrations (e.g., methylation silencing of DNA repair genes) could cause genetic instability, leading to carcinogenesis [11]. For example, somatic mutations in various epigenetic regulators (e.g., KDM6A, KMT2D, EZH2) in different tumor types have been found to induce aberrant DNA methylation profiles to promote cancer progression [12, 13, 14]. *TMPRSS2‐ERG* (T2E) fusion, as the most common genomic alteration in PC, is associated with distinct DNA methylation profiles, as opposed to T2E‐negative tumors [1, 15].

SNAI2 is one of three members of the Snail family of zinc finger transcription factors. It plays an important role in developmental biology by regulating adult stem and progenitor cell function and differentiation in different tissues [16]. For instance, SNAI2 promotes stem cell function and directs lineage specification through direct transcriptional repression of luminal differentiation genes [17]. SNAI2 is also a key regulator during epithelial‐to‐mesenchymal transition (EMT), which is an evolutionarily conserved transcriptional program and contributes significantly to tumor metastasis [18]. Strict regulation of *SNAI2* expression is essential for its key functions in different biological processes. Aberrant *SNAI2* expression has been observed in various cancer types and possibly predicts poor prognosis in cancer patients [19].

In this study, we unraveled that *SNAI2* is frequently amplified in PC, while its expression is significantly decreased. Further, T2E is involved in the epigenetic silencing of SNAI2, which is essential for aberrant cell proliferation and luminal differentiation. SNAI2 interacts with the tumor microenvironment by regulating reactive stroma and tumor‐infiltrating immune cell profiling. Importantly, epigenetic silencing of SNAI2 could induce resistance to the tyrosine kinase SRC inhibitor dasatinib in PC, while the histone deacetylase (HDAC) inhibitor LBH589 could enhance dasatinib resistance by increasing SNAI2 levels.

## 2 Materials and methods

### 2.1 Clinical cohort summary

Characteristics of PC patients in the Physicians' Health Study (PHS) and Health Professionals Follow‐up Study (HPFS) cohorts have been previously reported [20]. Unless otherwise specified, we will treat PHS/HPFS as one cohort for this report. An additional 16 publicly available PC cohorts are summarized in Table S1.

### 2.2 Cell culture and stable cell lines

The recourse of all cell lines is listed in Table S5. The ABL cell line was maintained in phenol red‐free RPMI1640 media supplemented with 10% CCS, 2 mm l‐glutamine, and 1 × antibiotic/antimycotic. RWPE1 cells were cultured in keratinocyte serum‐free medium (Thermo Fisher Scientific, Waltham, MA, USA). All other cell lines were maintained in 10% FBS supplemented with 2 mm of l‐glutamine and antibiotic at 37 °C in 5% CO_2_. Cells were authenticated by human short tandem repeat profiling at the MSK Integrated Genomics Operation Core Facility. Stable cells overexpressing SNAI2 were established as previously reported using SNAI2 expression and control vectors (Table S2) [20, 21].

### 2.3 RNA analysis, RNA sequencing, and immunoblotting

Total RNA isolation has previously been described [20]. TaqMan gene expression assays (Applied Biosystems, Thermo Fisher Scientific, San Francisco, CA, USA) were used for relative gene expression (Table S2) by qRT‐PCR. Transcript levels were normalized to levels of GAPDH transcript. RNA sequencing was performed by 50 million 2 × 50 bp reads in the MSK Integrated Genomics Operation Core. RNA sequencing data were analyzed using Partek Flow (Partek Inc., St. Louis, MO, USA). Proteins were extracted by RIPA buffer, and protein concentration was determined by the Bradford method. All antibodies used are listed in Table S2.

### 2.4 Cell viability and drug synergy assays

After cells were treated with inhibitors (Table S2) for 3–5 days, cell viability was assessed using the Cell Titer‐Glo luminescent cell viability assay (Promega Corp., Madison, WI, USA). Synergies between dasatinib and LBH589 were evaluated in LAPC4 and ABL cells after 5‐day treatment. SynergyFinder was used for the synergy effect analysis [22]. Distinct drug doses were applied in two cell lines because of the differing sensitivity. Drug combination responses were also plotted as heat maps to determine the therapeutic significance of the combination by identifying the concentrations at which the drug combination had maximum effect on PC cell growth inhibition. The degree of drug synergy was assessed using SynergyFinder (Tang Laboratory, University of Helsinki; https://synergyfinder.fimm.fi). Synergy assays were performed in triplicate. The summary synergy showed the average response to the drug combination. A synergy score of less than −10 was considered antagonistic, a range from −10 to +10 as additive, and greater than +10 as synergistic.

### 2.5 Methylation sequencing and data analysis

Genome‐wide DNA methylation profiling was performed using the Illumina TruSeq Methyl Capture EPIC library Prep Kit (Illumina, San Diego, CA, USA) and NGS technology for genomic DNA sequencing. Five hundred nanogram genomic DNA from four PC cells were used for the library preparation. An LE220‐plus Focused‐ultrasonicator (Covaris, Inc., Woburn, MA, USA) was used to shear 500 ng of genomic DNA. Sequencing libraries were prepared using the KAPA HyperPrep Kit (Roche Sequencing, Pleasanton, CA, USA). Postligation cleanup was performed using the TruSeq Methyl Capture EPIC LT Library Prep Kit (Illumina). After purification, samples were pooled; equimolar and methylome regions were captured using EPIC oligos and bisulfite, converted, and amplified with 11 cycles of PCR. Pools were sequenced on a HiSeq 4000 in a PE100 run, using the HiSeq 3000/4000 SBS Kit (Illumina).

To process methylation data from the epic methyl capture assay, we used the BISMARK package to map the bisulfite reads to the human genome. The bowtie2 aligner was used in the mapping step, and we used v0.23.0 of the BISMARK code. We followed the recommended workflow as outlined in github.com/FelixKrueger/Bismark/tree/master/Docs which consisted of the following steps: genome_preparation, Bismarck mapping, deduplication, and finally the quantity of the methylation signal with methylation_extractor. The data are available from GEO (GSE179214).

### 2.6 Bioinformatic analysis of clinical cohorts

Data for various clinical cohorts were obtained from cBioPortal for Cancer Genomics [23], KM plotter [24], and Oncomine [25]. Heatmaps were generated using Rv3.4.3 (https://www.R‐project.org). Pathway analysis was performed using gene set enrichment analysis (GSEA) [26]. Gene scores were calculated with gene set variation analysis using single‐sample GSEA (ssGSEA) [27]. The abundance of immune cell fractions in each sample was determined using cell type identification by estimating relative subsets of RNA transcripts (CIBERSORT) and LM22, a validated leukocyte gene signature matrix [28].

### 2.7 Statistical analysis

Results are reported as mean ± standard deviation. Comparisons between groups were performed using an unpaired two‐sided Student's *t*‐test or Wilcoxon rank‐sum test (*P* < 0.05 was considered significant). Disease‐free survival (DFS) was examined using the Kaplan–Meier method. Patients were divided into two groups (upper and lower quartile based on gene expression or gene signature score), and Kaplan–Meier curves were generated for each group. The log‐rank test was used to determine significance. Cox proportional hazard regression was performed, adjusting for clinical and demographic factors. Statistical analysis was completed using R version 3.4.3 (https://www.R‐project.org).

## 3 Results

### 3.1 Amplification and expression of *SNAI2* showed correlations with clinical outcomes in opposite manners

**Fig. 1:**
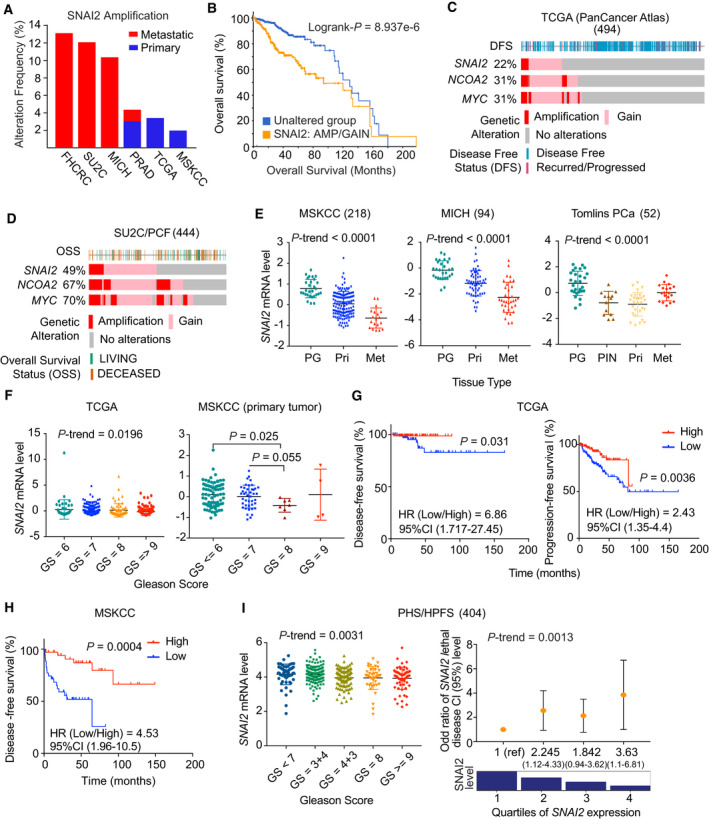
Amplification of *SNAI2* is correlated with poor clinical outcomes in prostate cancer. (A) Amplification of *SNAI2* in six PC cohorts. The data were extracted from cBioPortal. (B) Correlation between *SNAI2* genomic alteration and OS in the TCGA cohort. (C, D) The genomic alteration of *SNAI2*, NCOA2, and MYC in the TCGA (C) and SU2C/PCF (D) cohorts. (E) Correlation between *SNAI2* levels and tumor progression in three cohorts. (F–H) Correlation between *SNAI2* levels and clinical outcomes, including Gleason grades (F), DFS or PFS (G, H) in the TCGA and MSKCC cohorts. (I) Correlation between *SNAI2* levels and Gleason grades and the risk of lethal PC over long‐term follow‐up in the combined HPFS and PHS cohorts. Patient numbers from each cohort are listed in Table S1. Figure values represent the mean ± SD. The *P*‐value was calculated by Wilcoxon rank‐sum test. Time‐to‐event outcomes were analyzed using the Kaplan–Meier method and compared via the log‐rank test.

In six PC clinical cohorts, amplification of *SNAI2* was frequently observed in both primary (4%) and metastatic (13%) disease (Fig. 1A). Copy number alteration (CNA) of *SNAI2*, by either amplification or gain, was significantly associated with worse overall survival (OS) in six PC cohorts (Fig. 1B). However, in some PC cohorts, CNA was observed co‐occurring in *SNAI2* and its neighbor genes (e.g., *NCOA2* and *MYC*; Fig. 1C,D, Fig. S1A,B). The CNA of *NCOA2* and *MYC* was significantly correlated with worse OS in six PC cohorts (Fig. S1C). These suggested that whole‐arm amplification of chromosome 8q could contribute to the clinical significance, instead of a single gene like *SNAI2*.

To determine the clinical significance of *SNAI2*, we analyzed the correlation between *SNAI2* expression levels and clinical outcomes. In nine cohorts of PC patients, *SNAI2* expression was significantly decreased in tumor tissue compared with normal prostate tissue (Fig. 1E, Fig. S2A,B), and *SNAI2* levels were significantly reduced in primary tumors of higher Gleason grade (Fig. 1F, Fig. S2C). Furthermore, in the TCGA and MSKCC cohorts, low *SNAI2* levels were significantly associated with worse DFS and progression‐free survival (PFS) (Fig. 1G,H). We further validated the prognostic significance of *SNAI2* levels using the HPFS/PHS (*n* = 150), two publicly unavailable cohorts with long‐term follow‐up for fatal outcomes. Significantly lower levels of SNAI2 in these cohorts were correlated with high Gleason score (*P*_trend_ = 0.0031; Fig 1I, left) and an increased risk of lethal disease (Fig. 1I, right). *SNAI2* RNA levels in the lowest quartile, compared with the highest quartile, were associated with a 3.63 times higher risk of lethal disease (95% CI, 1.1–6.81). Furthermore, SNAI2 protein expression is highly correlated with DFS and PFS in TCGA, but MYC and NCOA2 do not show a significant correlation (Fig. S2D–F).

We analyzed the correlation between *SNAI2* levels and OS in 19 cancer types using Kaplan–Meier plotting (Table S3) [24]. High *SNAI2* expression was significantly correlated with worse OS in most cancer types—except uterine corpus endometrial carcinoma, in which low *SNAI2* was correlated with worse OS. Altogether, reduced *SNAI2* expression may contribute to initiation and progression of PC.

### 3.2 DNA methylation regulates *SNAI2* expression in PC

**Fig. 2:**
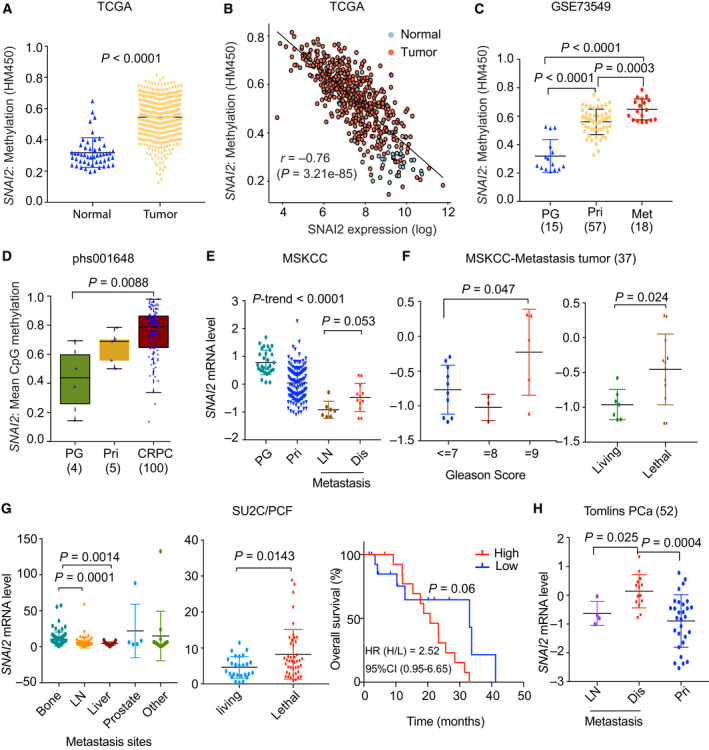
Methylation silencing of *SNAI2* occurs in PC, and reactivated SNAI2 in distant metastatic tumors is correlated with poor clinical outcomes. (A) Methylation status of *SNAI2* in normal tissue and primary PC in the TCGA cohort. (B) Anti‐correlation of methylation and mRNA levels of *SNAI2* in TCGA. (C, D) Methylation status of *SNAI2* in two cohort datasets (GSE73549, phs001648). The methylation data from phs001648 were acquired from the whole‐genome bisulfite sequencing and mean CpG methylation levels were calculated. (E) *SNAI2* levels across normal tissue and various tumor types in the MSKCC cohort. (F) Correlation between *SNAI2* levels and Gleason grades (left) and lethality (right) in metastatic tumors in the MSKCC cohort. (G) Correlation between *SNAI2* levels and metastatic sites (left), lethality (middle), and OS (right) in metastatic tumors in the SU2C/PCF cohort. (H) Correlation between *SNAI2* levels and tumor types in the Tomlins PC cohort. Patient numbers from each cohort are listed in Table S1. Figure values represent the mean ± SD. The *P*‐value was calculated by Wilcoxon rank‐sum test. Time‐to‐event outcomes were analyzed using the Kaplan–Meier method and compared via the log‐rank test. CRPC, castration‐resistant prostate cancer; Dis, distant metastasis; LN, lymph node metastasis; PG, prostate gland; Pri, primary tumor.

The inconsistent prognostic significance between CNA and mRNA expression of *SNAI2* led us to analyze the correlation between CNA and mRNA expression of *SNAI2* in PC. CNA of *SNAI2* was not correlated with *SNAI2* RNA expression in multiple PC cohorts (Fig. S3A,B), indicating that epigenetic regulation of *SNAI2* may contribute to SNAI2 silencing. Methylation of the *SNAI2* gene in PC has been reported [29]. We confirmed that methylation of *SNAI2* was significantly increased in primary prostate tumors compared to normal prostate tissue and showed negative correlation with *SNAI2* mRNA in TCGA (*r* = −0.73, *P* = 3.21e‐85) (Fig. 2A,B). Additionally, the high methylation and low expression of *SNAI2* were positively associated with higher fraction genome alteration (FGA) (Fig. S3C,D). Methylation of *SNAI2* was detected not only in primary tumors but also in metastatic tumors (Fig. 2C,D) [10, 30]. It was reported that low expression of SNAI2 was found in most PC tissue, but higher SNAI2 expression was detected only in the cancer cell clusters at the invasion/expansion front [29]. Given the oncogenic function of SNAI2 in tumor invasion, we analyzed the SNAI2 levels in defined metastasis types. Intriguingly, in the MSKCC cohorts, a relatively higher level of SNAI2 was observed in distant metastatic tumors than in lymph node metastases (Fig. 2E). Among the metastatic tumors, significantly higher levels of SNAI2 were associated with higher Gleason grade (Gleason Score = 9) and lethality (Fig. 2F). These results were confirmed in the SU2C/PCF cohort, which only contains metastatic tumors. *SNAI2* levels were higher in bone metastases than in liver and lymph node metastases (Fig. 2G, left) and were significantly associated with lethality and OS (Fig. 2G, right). In the Tomlins cohort, *SNAI2* levels were higher in distant metastases than in both lymph node metastases and primary tumors (Fig. 2H). Together, these results show that methylation of *SNAI2* contributes to decreased *SNAI2* expression, and reactivating SNAI2 may be required for metastatic tumor progression.

### 3.3 *TMPRSS2‐ERG* (T2E) fusion regulates silencing of *SNAI2* in PC

**Fig. 3:**
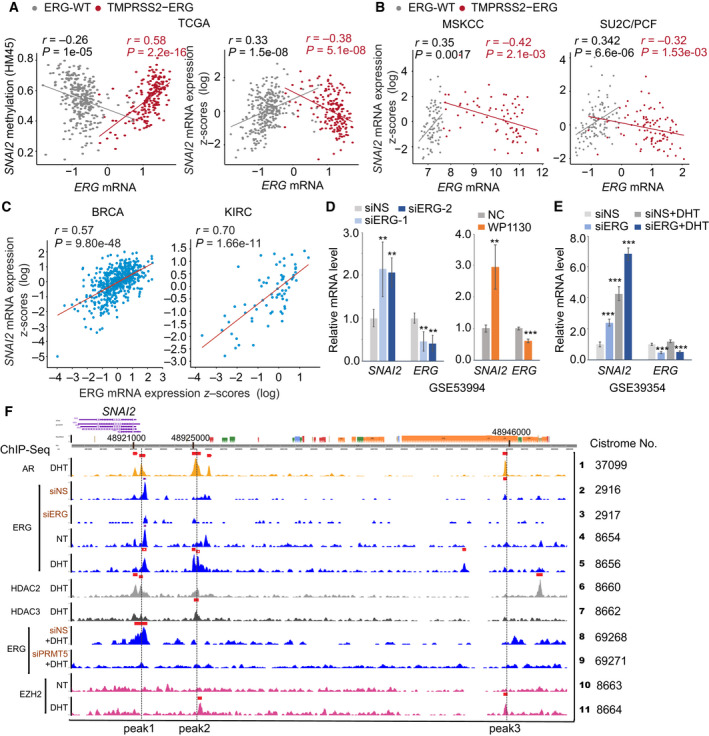
TMPRSS2‐ERG is involved in the epigenetic silencing of *SNAI2* in PC. (A) Correlation between *ERG* expression and methylation and mRNA levels of *SNAI2* in the TCGA cohort. (B) Correlation between *ERG* expression and mRNA levels of *SNAI2* in the MSKCC and SU2C/PCF cohorts. (C) Correlation between *ERG* expression and mRNA levels of *SNAI2* in the BRCA and KIRC cohorts. (D, E) The effects on *SNAI2* expression by inhibition of ERG by siRNAs or WP130 in VCaP cells. The datasets from GSE53994 (D) and GSE39354 (E) were used for the analysis. (F) Transcription factors and histone modifiers binding to *SNAI2* in VCaP cells. The multiple ChIP‐Seq datasets were extracted from the Cistrome Data Browser. Figure values represent the mean ± SE of duplicates. Comparisons between groups were performed using an unpaired two‐sided Student's *t*‐test. **P < 0.01; ***P < 0.001; vs. control groups treated with nonspecific (siNS) siRNA or DMSO.

Several studies demonstrated that T2E, as the earliest common fusion event in PC, is correlated with distinct methylation profiles of wild‐type *ERG* (ERG‐WT) [1, 15]. We found that ERG overexpression induced by T2E was positively correlated with *SNAI2* methylation (*r* = 0.58, *P* = 2.2e‐16) and negatively correlated with *SNAI2* mRNA expression (*r* = −0.38, *P* = 5.1e‐08) (Fig. 3A). ERG‐WT tumors exhibited the opposite correlation patterns to T2E tumors. This finding was confirmed in the MSKCC and SU2C/PCF cohorts (Fig. 3B).

T2E tumors also exhibited distinct correlation patterns with other *ERG* targets (e.g., *CACNA1D* and *NT5C*, which are regulated by *ERG* differently [15] (Fig. S4A–D). ERG‐WT expression is neglected, but T2E induces ERG overexpression in PC [31]. To investigate if *ERG* levels lead to the distinct regulation patterns seen in T2E and ERG‐WT tumors, we assessed *ERG* levels across multiple cancer types (Fig. S4E). Compared with PC (PRAD cohort), *ERG* levels were higher in kidney cancer (KICH cohort) and comparable in breast cancer (BRCA cohort). In both the BRCA and KICH cohorts, *ERG* expression was positively correlated with *SNAI2* expression (Fig. 3C), consistent with the reported transcriptional activation role of ERG [32]. We further confirmed this result in multiple cancer types (Table S4). Taken together, these results suggest that the distinct regulation of *SNAI2* in T2E tumors may be specific to T2E fusion alteration but not due to high *ERG* expression.

The repression of *SNAI2* by T2E was validated in two datasets in which T2E in VCaP cells was inhibited by either siERG or small molecule (WP1130) [33, 34]. *SNAI2* expression was significantly increased in VCaP cells (Fig. 3D,E), while two other reported ERG targets (*PLA1A* and *KCNS3*) were downregulated due to positive regulation by T2E (Fig. S4F–H). ChIP‐Seq datasets from four studies confirmed the transcriptional or epigenetic repression of *SNAI2* by T2E [35, 36, 37, 38]. ERG was proven to bind to the AR binding sites [38], and knockdown of T2E by siERG in VCaP cells abolished ERG‐specific binding (Peak 1–3) (Fig. 3F), which overlapped with AR binding to the *SNAI2* promoter and enhancer (Fig. 3F, lanes 1–5). We also observed repressive epigenetic modifiers (e.g., HDAC2, HDAC3, and EZH2) binding to the same regions with dihydrotestosterone (DH) treatment (Fig. 3F, lanes 6, 7, 10, 11). Intriguingly, histone methyltransferase, PRMT5 and EZH2, showed distinct binding preference to three ERG‐binding regions (Fig. 3F, lanes 8–11). These findings demonstrated that T2E could repress SNAI2 expression by recruiting multiple modifiers to SNAI2 gene.

### 3.4 Identify the potential epigenetic regulators of *SNAI2*

**Fig. 4:**
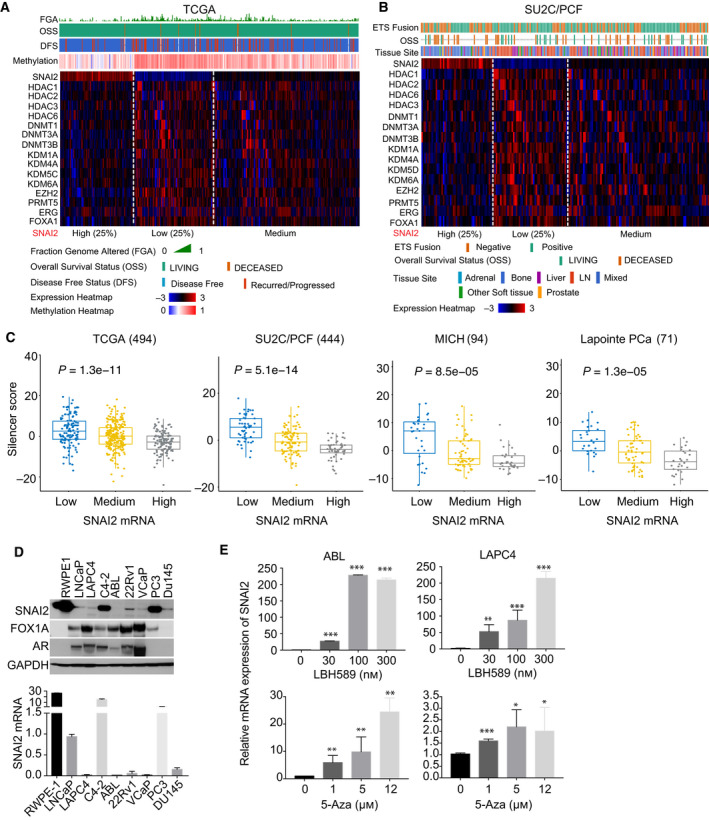
Methylation regulation of *SNAI2* in clinical cohorts and PC cell lines. (A, B) Profiles of potential epigenetic silencers of SNAI2 in the TCGA (A) and SU2C/PCF (B) cohorts. The heat maps integrated clinical attributes with epigenetic silencers profiling, which was extracted from cBioPortal. (C) The correlation of SNAI2 levels with Silencer score in four cohorts. The Silencer score of potential epigenetic silencers was created by using ssGSEA. Comparisons between groups were performed by Wilcoxon rank‐sum test. (D) SNAI2 protein and mRNA levels across multiple PC cell lines. (E) Effects of DNMTi (5‐Aza, 5‐Aza‐2′‐deoxycytidine) and HDACi (LBH589) treatment on SNAI2 levels in ABL and LAPC4 cells. The SNAI2 mRNA levels were detected after 5 days of treatment with 5‐Aza or 1 day of treatment with LBH589. Patient numbers from each cohort are listed in Table S1. Figure values represent the mean ± SE of three independent experiments. Comparisons between groups were performed using an unpaired two‐sided Student's *t*‐test. *P < 0.05; **P < 0.01; ***P < 0.001; vs. control groups treated with DMSO.

Multiple epigenetic modifiers showed anti‐correlation with *SNAI2* expression in PC cohorts. They regulate histone deacetylation (HDAC1, 2, 3, 6), DNA methylation (DNMT1, 3A, 3B), histone methylation (KDM1A, ‐4A, ‐5C, ‐6A, EZH2, PRMT5), and transcriptional repressors (ERG, FOXA1), but *SNAI2* expression is not correlated with AR expression in either primary (TCGA) or metastatic PC tumors (SU2C and FHCRC) (Fig. S5A). Accordingly, their upregulation is significantly associated with clinical outcomes (FGA signal, OSS, DFS) in both the TCGA and SU2C cohorts (Fig. 4A,B, Fig. S5B–F). The Silencer score was created to quantify the correlations of these epigenetic modifiers with SNAI2 expression. SNAI2‐low samples had a high Silencer score in four cohorts (Fig. 4C). Next, we found that SNAI2 expression was very low in most PC cell lines, except PC3 and C4‐2, compared to normal prostate cells (e.g., RWPE‐1) (Fig. 4D, Fig. S5G). Methylation sequencing analysis showed significant higher methylation signal of SNAI2 in the cell lines which express very low SNAI2, including ABL, LAPC4, and VCaP cells (Fig. S5H). DNMT inhibitor (5‐Aza) and HDAC inhibitor (LBH589) effectively restored *SNAI2* expression in PC cells (Fig. 4E, Fig. S5I). In addition, LBH589 treatment did not change MYC and NCOA2 gene expression in ABL and LAPC4 cells (Fig. S5J). LBH589 showed more potent activation activity than 5‐Aza by increasing *SNAI2* levels at 15‐250‐fold, indicating transcriptional repression besides DNA methylation controls *SNAI2* expression.

### 3.5 Silencing of SNAI2 may be essential for robust tumor cell proliferation in PC

**Fig. 5:**
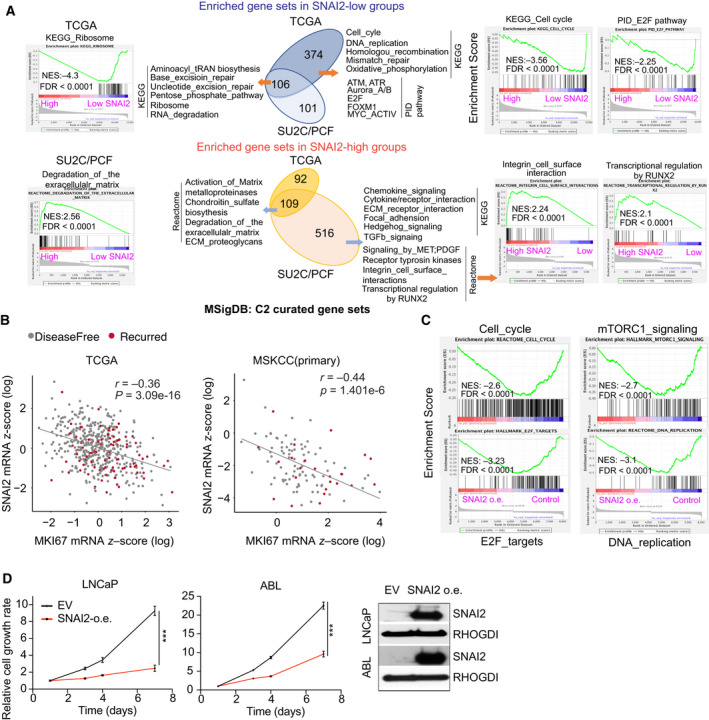
Silencing of SNAI2 is required for tumor cell proliferation. (A) Comparison of highly enriched pathways in *SNAI2*‐high and *SNAI2*‐low groups in primary (TCGA) and metastatic (SU2C/PCF) cohorts. MSigDB: C2 gene sets were used for GSEA. (B) Correlation between *SNAI2* and *MKI67* levels in the TCGA and MSKCC cohorts. (C) Enrichment of cell proliferation‐related pathways in PC cells overexpressing SNAI2. The dataset was extracted from GSE80042. (D) The effects on cell proliferation of PC cells overexpressing SNAI2. Cell viability was detected in 7 days. SNAI2 expression was detected by immunoblotting. Figure values represent the mean ± SE of three independent experiments. Comparisons between groups were performed using an unpaired two‐sided Student's *t*‐test. ***P < 0.001; vs. control groups infected with empty vector (EV).

To investigate why SNAI2 is silenced in PC, we applied GSEA to genes highly enriched for either low or high SNAI2 levels in both the TCGA (primary tumor) and SU2C (metastatic tumor) cohorts. The common enriched gene sets in groups with low SNAI2 in those two cohorts are in the ribosome, excision repair, and tRAN synthesis pathways (Fig. 5A). In low‐SNAI2 groups in the TCGA cohort, 374 gene sets were highly enriched in the cell cycle, DNA replication, DNA repair, and energy metabolism pathways, which support cell proliferation. Pathway Interaction Database pathway analysis also confirmed the enrichment of multiple cell cycle‐regulated pathways in groups with low SNAI2 in the TCGA cohort (Fig. 5A, upper panel). In metastatic PC, groups with high SNAI2 had worse clinical outcomes (Fig. 2D,E). In the SU2C cohort, 516 gene sets were exclusively enriched in the high‐SNAI2 group; these gene sets play important roles in tumor invasion and metastasis, such as focal adhesion, hedgehog, MET, PDGFP, and integrin signaling (Fig. 5A, lower panel). RUNX2‐regulated targets were also highly enriched in the 516 gene sets. *RUNX2* gene expression showed negative correlation with *SNAI2* genes in primary and metastatic tumors (Fig. S6A). The 109 common gene sets in both cohorts were mainly enriched in matrix degradation pathways (Table S5). These findings demonstrate the oncogenic function of SNAI2 to promote tumor invasion.

As the cell proliferation index, *MKI67* showed anti‐correlation with SNAI2 levels in primary PC (Fig. 5B), but not in metastatic PC (Fig. S6B). In the SNAI2‐inducible cell line models created by Stylianou *et al*. [39], SNAI2 was overexpressed with doxycycline induction, while SNAI2 levels decreased substantially when doxycycline was withdrawn for 20 days. We applied GSEA analysis using datasets from their study. Consistent with the clinical cohort data, pathways related to cell proliferation (e.g., cell cycle, E2F targets, mTOR signaling, and DNA replication) were inhibited, but hallmark of EMT and angiogenesis were activated in cells overexpressing SNAI2 (Fig. 5C, Fig. S6C). The opposite results were observed in SNAI2 knockdown cells (Fig. S6D). In our PC cell lines stably overexpressing SNAI2 (LNCaP, ABL, and 22Rv1), cell proliferation was significantly inhibited (Fig. 5D, Fig. S6E), while overexpression of SNAI2 induced remarkable cell invasion ability (Fig. S6F). Thus, we demonstrated that silencing SNAI2 contributes to cell proliferation.

### 3.6 Silencing of SNAI2 could contribute to luminal differentiation in PC

**Fig. 6:**
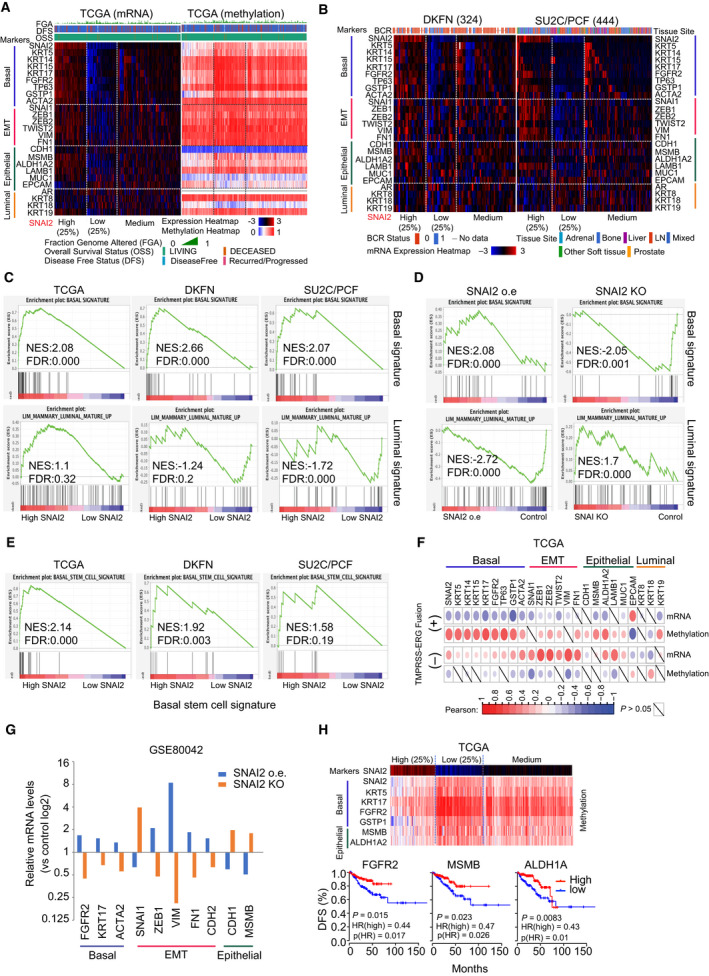
Silencing of SNAI2 contributes to luminal differentiation in PC. (A, B) Lineage marker profiling in three PC cohorts (TCGA, DKFN, and SU2C/PCF). The heat maps integrated clinical attributes with marker profiling, which was extracted from cBioPortal. (C, D) Basal and luminal signature analysis in clinical cohorts (C) and cell line models (with SNAI2 overexpression (o.e.) or SNAI2 knockdown) (D). (E) Basal stem cell signature analysis in three clinical cohorts. (F) Correlation between ERG expression and methylation and mRNA levels of lineage markers in the TCGA cohort. (G) The effects on lineage marker expression by SNAI2 overexpression or knockdown in PC cells. The dataset was extracted from GSE80042. (H) Methylation profiling of lineage markers in the TCGA cohort. Three genes of the markers showed correlation with DFS. Time‐to‐event outcomes were analyzed using the Kaplan–Meier method and compared via the log‐rank test. Patient numbers from each cohort are listed in Table S1.

The imbalance of the differentiation process leads to the accumulation of hyper‐proliferative differentiated luminal cancer cells [40, 41]. SNAI2 is highly expressed in basal cells, and inhibition of SNAI2 can increase luminal cell population [17]. A panel of lineage markers was assessed in three cohorts (TCGA, DKFN, and SU2C/PCF). In TCGA and DKFN, all markers except luminal markers and methylation profiling were highly concurrent with SNAI2 (Fig. 6A,B). Low levels of SNAI2 were significantly associated with high disease stages and Gleason grades in the DKFN cohort (Fig. S7A,B). Intriguingly, most of the basal and epithelial markers are expressed at low levels in metastatic patient tissues with high SNAI2 expression, while only EMT markers are highly expressed, which supports the aggressive nature of these tumors (Figs 2E and 6B right). Basal signature was enriched in patient tissues with high SNAI2 levels and in cells overexpressing SNAI2 (Fig. 6C) [42], while luminal signature was enriched in patient tissues with low *SNAI2* levels and in SNAI2 knockdown cells (Fig. 6D). Basal PC cells show stem cell properties [43]; we found the basal stem cell signature was also highly enriched in patient tissues with high *SNAI2* (Fig. 6E).

Similar to *SNAI2*, in both the TCGA and MSKCC cohorts, the methylation status and mRNA level of most markers tested showed significant correlations with T2E (Fig. 6F, Fig. S7C). However, these correlations were remarkably attenuated in the SU2C cohort of metastatic prostate cancer (Fig. S7C). As a transcriptional factor, SNAI2 directly regulates these markers in SNAI2‐inducible cell models (GSE80042) (Fig. 6G) [39]. Two luminal markers (KRT18 and KRT19) were significantly repressed by SNAI2 overexpression (Fig. S7D). Seven basal and epithelial markers shared the same methylation profile with *SNAI2* (Fig. 6H, upper). Three of them showed significant correlations with DFS in the TCGA cohort (Fig. 6H, lower). High levels of *FGFR2* were correlated with better DFS in TCGA (Fig. 6H), but with worse OS in SU2C/PCF (Fig. S7E), due to its role in metastasis [44]. High *MSMB* maintained the association with better DFS (in TCGA) and OS (in SU2C/PCF) (Fig. 6H, Fig. S7E). None of the other markers in the panel showed association with clinical outcomes (Fig. S7F). These results suggest that T2E, like *SNAI2*, may be involved in the regulation of a panel of lineage markers supporting tumor initiation and expansion in PC.

### 3.7 SNAI2 interacts with the tumor microenvironment in PC

**Fig. 7:**
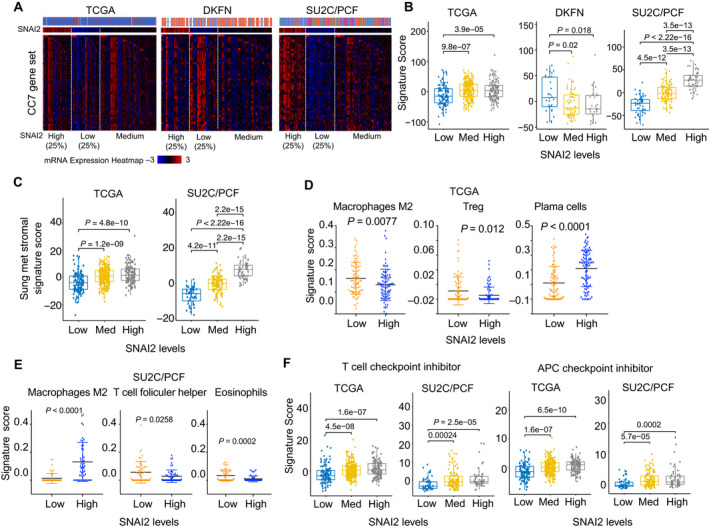
SNAI2 interacts with the tumor microenvironment in PC. (A) Correlation between *SNAI2* levels and reactive stromal signature (CC7) profiling in three clinical cohorts. (B) Correlation between *SNAI2* levels and CC7 signature score in the same three clinical cohorts. (C) Correlation between *SNAI2* levels and Sung metastasis stromal signature score in the TCGA and SU2C cohorts. (D, E) Profiling of immune cells by CIBERSORT in the TCGA (D) and SU2C/PCF (E) cohorts. (F) Correlation between *SNAI2* levels and checkpoint inhibitor signatures in the TCGA and SU2C/PCF cohorts. T‐cell checkpoint inhibitor and APC checkpoint inhibitor signatures were applied for ssGSEA analysis. Significance was determined using Wilcoxon's rank‐sum test with Benjamini–Hochberg correction. Figure values represent the mean ± SD.

The interplay of the tumor, stromal cells, and immune system constitutes the tumor microenvironment [45, 46]. The oncogenic function of SNAI2 in PC metastasis is well established [47, 48]; e.g., SNAI2 reactivates tumor stroma to facilitate tumor metastasis [49]. Gerhauser *et al*. [41] identified seven distinct CLICK clusters (abbreviated CC1–7) to distinguish the molecular subgroups associated with disease progression. Cluster CC7, which is enriched with a reactive stroma signature, is significantly correlated with worse clinical outcomes. We assessed the correlation between *SNAI2* expression and CC7 signature in PC cohorts. Among 86 CC7 genes, 42 are overlapped with genes highly enriched in groups with high *SNAI2* levels in the TCGA and SU2C cohorts (Fig. S8A). The heat map showed stronger correlation of these 42 CC7 genes with *SNAI2* in SU2C than in TCGA (Fig. 7A). Surprisingly, 42 CC7 genes are highly expressed in the low‐SNAI2 group in the DKFN cohort (Fig. 7A), which is clinically significant. The group with low SNAI2 in the DKFN cohort harbored a similar aggressive potential as the group with high SNAI2 in SU2C, although DKFN cohort includes only primary tumors. This is consistent with the fact that patients with early‐onset PC (in DKFN) are likely to develop a severe disease course [41]. The ssGSEA with 42‐CC7 signature provided quantitative evidence (Fig. 7B). We confirmed this finding by using two other metastatic stromal signatures (Fig. 7C, Fig. S8C) [50, 51].

Next, CIBERSORT was used to assess the correlation between SNAI2 levels and the tumor immune microenvironment [28]. In the TCGA cohort, immunosuppressive regulatory T cells (Tregs) (*P* = 0.012) and M2 macrophages (*P* = 0.0077) were more abundant in tumors with low SNAI2 levels, whereas the infiltration of antitumor immune plasma cells was significantly lower in the low‐SNAI2 group than in the high‐SNAI2 group (Fig. 7D). In the SU2C/PCF cohort, tumors with high SNAI2 had significantly more immunosuppressive M2 macrophages and less antitumor eosinophils than tumors with low SNAI2 levels (Fig. 7E). The signature scores of the other types of immune cells with significant difference in these cohorts are shown in Fig. S8D,E. Therefore, *SNAI2* expression is correlated with the immunosuppressive tumor immune microenvironment in different manners in primary and metastatic PC, which supports the distinct clinical relevance of SNAI2 expression at different stages of the disease.

### 3.8 SNAI2 levels is correlated with dasatinib sensitivity

**Fig. 8:**
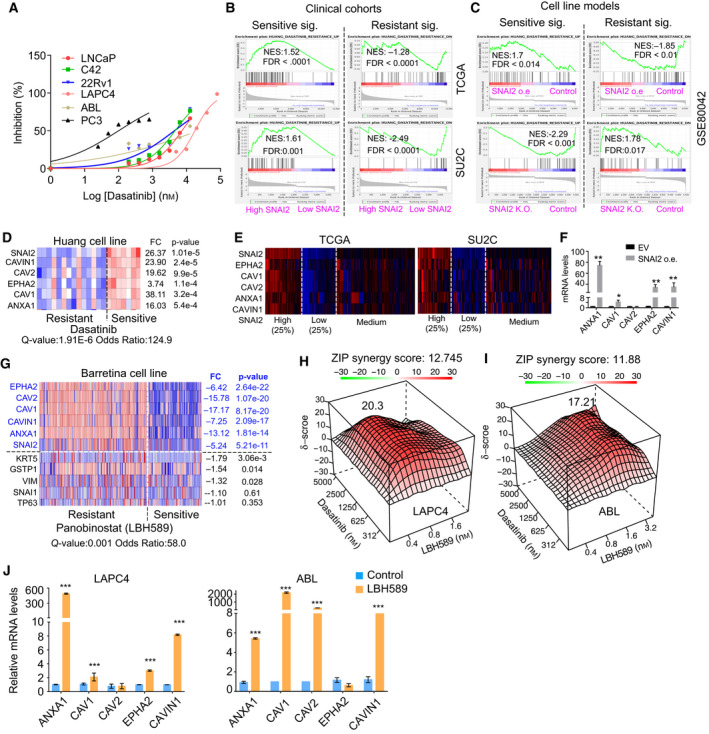
SNAI2 levels determine dasatinib sensitivity. (A) Dasatinib sensitivity in six PC cell lines. Cell viability was detected after dasatinib treatment for 5 days. (B, C) Dasatinib‐sensitive and resistant signature enrichments in clinical cohorts (B) and cell line models (with SNAI2 overexpression or knockdown) (C). Dasatinib response signatures were used for GSEA analysis using datasets extracted from TCGA/SU2C cohorts and GSE80042. (D, E) 5‐gene signature plus *SNAI2* profiling in breast cancer (Huang cell lines) (D) and PC (TCGA, SU2C) cohorts (E). (F) Regulation of a 5‐gene signature by overexpressing SNAI2 in ABL cells. Gene expression was detected by qRT‐PCR. (G) The dasatinib‐sensitive signature profiling in a Barretina cell line dataset. Three basal markers (KRT5, GSTP1, TP63) and two EMT markers (VIM, SNAI1) were used as negative control of dasatinib‐sensitive signature in the profiling. (H, I) Synergy assays for the combination of dasatinib and LBH589 in LAPC4 (H) and ABL (I) cells. Cells' viability was detected after 5 days of treatment with drugs. SynergyFinder was used to calculate the ZIP synergy score. ZIP > 10 suggested a synergistic effect. (J) The effects of LBH589 treatment on a 5‐gene signature in LAPC4 and ABL cells. Gene expression was detected after one day of LBH589 treatment by qRT‐PCR. Figure values represent the mean ± SE of three independent experiments. *P < 0.05; **P < 0.01; ***P < 0.001 vs. control groups infected with empty vector (EV) or treated with DMSO.

In the TCGA cohort, tyrosine kinase signaling‐related proteins are highly enriched in high SNAI2 groups (Fig. S9A), indicating that SNAI2 level could be correlated with tyrosine kinase activity. Analysis of a dataset (GSE9633) reporting dasatinib sensitivity among PC cells showed that dasatinib‐resistant cells exhibited a remarkably lower SNAI2 level (Fig. S9B) [52]. We confirmed that PC3 cells with the highest SNAI2 level showed the highest sensitivity to dasatinib, compared to other PC cells with low expression of SNAI2 (Figs 4D and 8A). Dasatinib response signatures that were developed in 23 breast cancer cell lines were applied to PC cohorts and cell models [53]. A dasatinib‐sensitive signature was highly enriched in PC tissue with high SNAI2 and cells overexpressing SNAI2, while a dasatinib‐resistant signature was enriched in tissue with low SNAI2 and SNAI2 knockdown cells (Fig. 8B,C). The dasatinib‐sensitive signature can be simplified as a 6‐gene signature [53], five of which are highly correlated with *SNAI2* levels in three cell line datasets (Fig. 8D, Fig. S9C,D) [52, 53, 54]. These observations were confirmed in both the TCGA and SU2C cohorts (Fig. 8E). Intriguingly, the five genes (*CAVIN1, EPHA2, CAV2, CAV1*, *ANXA1*) were directly regulated by overexpressing SNAI2 (Fig. 8F, Fig. S9E), suggesting SNAI2 is the driver of dasatinib sensitivity. Overexpressing SNAI2 in LNCaP significantly increased the sensitivity to dasatinib, compared to untreated control cells (Fig. S9F), while knockdown of SNAI2 reduced dasatinib sensitivity in both PC3 and C4‐2 cells in which SNAI2 levels were high (Fig. S9G,H).

In the Barretina cell line dataset, 250 cancer cell lines were used to test panobinostat (LBH589) sensitivity [55]. All the LBH589‐sensitive cells showed significant low expression of dasatnib‐sensitive signature and SNAI2 (Fig. 8G). Other basal genes (*TP63*, *GSTP1*, *KRT5*) and EMT genes (*SNAI1, VIM*) showed much less or no significance to LBH589 sensitivity (Fig. 8G). Consistently, PC3 cells, as the dasatinib‐sensitive PC cells, show the least sensitivity to LBH589 (Fig. S9I). We revealed LBH589 significantly induced SNAI2 expression (Fig. 4E), suggesting that LBH589 may change dasatinib resistance. Synergistic effects between dasatinib and LBH589 were evaluated in LAPC4 and ABL cells, which are resistant to dasatinib. SynergyFinder analysis showed strong synergistic effects in the combination of drugs. The average of ZIP synergy score for dasatinib is 12.75 in LAPC4 and 11.88 in ABL, and the most synergistic area score is 20.3 (LAPC4) and 17.2 (ABL) (Fig. 8H,I). In LAPC4, 2.5 µm dasatinib induced a 3.35% cell growth inhibition, while 1.6 nm LBH589 induced 2.99% growth inhibition. The combination of these two doses inhibited cell growth 27.24% (Fig. S9H, left). These results were confirmed in ABL cells (Fig. S9I, right). In addition, LBH589 treatment significantly induced expression of the dasatinib‐sensitive gene signature, which could be the mechanism behind the synergistic effects (Fig. 8J). Altogether, we demonstrated that SNAI2 level affects dasatinib sensitivity, and LBH589 can enhance dasatinib sensitivity by increasing SNAI2 and other 5‐gene dasatinib‐sensitive signatures in PC.

## 4 Discussion

Genomic and epigenomic alterations collaboratively contribute to the heterogeneity seen in PC. In this study, we defined the mechanisms behind the silencing of SNAI2 in PC, which provided a perfect example of how the crosstalk between genomic and epigenomic alterations control PC initiation and progression. We revealed that T2E is involved in the silencing of SNAI2 and may be essential for aberrant tumor cell proliferation and luminal differentiation. Importantly, for the first time, we unraveled that SNAI2 levels can determine sensitivity to dasatinib.

SNAI2 requires strict regulation of its expression and activity in tissues, given its broad biological functions [56]. Our analysis suggests that SNAI2 expression holds distinct clinical significance at different stages of PC, indicating a dynamic change of SNAI2 levels during disease initiation and progression (Fig. 2A–H). The hallmark of primary tumors is highly proliferative, which is supported by silencing of SNAI2. However, the hallmark of metastatic tumors is highly invasive with slow proliferation, supported by activation of SNAI2. The SNAI2‐inducible cell lines established by Stylianou *et al*. [39] were ideal cell models to investigate SNAI2 function during different disease stages. SNAI2 overexpression was induced for 5 days to mimic the EMT process (which represented metastasis initiation), while SNAI2 expression was silenced for 20 days to mimic the MET (mesenchymal–epithelia–transition) process (which represented colonization after successful metastatic dissemination). GSEA analysis supported our hypothesis that silencing of SNAI2 was essential for cell proliferation and activation of SNAI2‐promoted cell invasion.

Our clinical cohort analysis showed relatively higher SNAI2 levels in distant metastatic tissues than in lymph node metastases, suggesting the dynamic changes in SNAI2 expression during tumor progression. Esposito *et al*. [29] reported reactivated SNAI2 at the edge of high‐grade PC tumors by IHC staining, suggesting that reactivation of SNAI2 occurs in only a small proportion of the cell population, which is sufficient for development of distant metastases. Methylation of SNAI2 is strongly maintained in metastatic PC (Fig. 2C), and T2E‐repressing *SNAI2* may be required for colonization of metastatic cells, as T2E was negatively correlated with SNAI2 expression in the SU2C/PCF (metastatic) cohort. RUNX2‐related genes were highly enriched in the group with high *SNAI2* in SU2C (Fig. 5A). The transcriptional repression of *SNAI2* by FOXA1 could be attenuated by the reduction of FOXA1 in metastatic PC [57]. RUNX2, as the key transcription factor in bone development, transcriptionally activates *SNAI2* [58]. *RUNX2* showed significant positive correlation with *SNAI2* expression in PC clinical cohorts (Fig. S6A), indicating RUNX2 could be a driving force in activating SNAI2 during the development of bone metastasis. AR and RUNX2 could coactivate SNAI2 in PC cells, but in clinical cohorts, only positive correlation was observed between RUNX2 and SNAI2. Furthermore, AR is highly expressed in luminal‐type PC cells, but SNAI2 is expressed in basal‐type PC cells. Interestingly, a recent study has reported that deficiency of SNAI2 in PC patients is correlated with better response to AR‐targeting therapies [59]. Therefore, whether and how AR regulates SNAI2 in PC patients remains to be determined. Epigenetic control of transcriptional regulation could determine dynamic change of SNAI2 expression to contribute to PC initiation and progression.

Based on our understanding of the mechanisms and function of SNAI2 silencing in PC, our further study revealed that SNAI2 level is the key to dasatinib sensitivity. The 5‐gene dasatinib‐sensitive signature was a direct target of SNAI2 in PC. There are 15 clinical trials evaluating dasatinib efficacy in PC. Although the benefit of using dasatinib for bone‐related disease has been confirmed, PC patients generally do not respond well to this agent. Our study proposed a mechanism of resistance: SNAI2 silencing in most cases of PC could cause the lack of response to dasatinib. We found that LBH589 effectively enhances dasatinib sensitivity at least partially by markedly increasing *SNAI2* levels.

Intriguingly, we revealed that cancer cells resistant to dasatinib are sensitive to LBH589. A phase II clinical trial using a combination of LBH589 and bicalutamide, an AR inhibitor, in patients with castration‐resistant PC showed significantly better PFS than with either drug alone [60]. Significant activation of *SNAI2* by LBH589 could promote progression in castration‐resistant disease. Our finding may provide a potential therapeutic strategy to prevent this disadvantage by adding dasatinib to the HDAC and AR inhibitor combination.

## 5 Conclusion

In summary, we integrated clinical cohort analysis with experimental validation to elucidate: (a) the distinct clinical relevance of SNAI2 at different disease stages; (b) that T2E‐regulated epigenetic silencing may contribute to dynamic changes in SNAI2 levels in PC; (c) that silencing of SNAI2 is required for cell proliferation and luminal differentiation; (d) that SNAI2 interacts with the tumor microenvironment by activating stroma and increasing immunosuppressive immune cell abundance; (e) that restoring SNAI2 expression by HDAC inhibition reverses dasatinib resistance. Our findings proposed a drug resistance mechanism and developed a novel strategy to increase the benefit of dasatinib in patients with PC.

## Conflict of interest

As of August 17, 2021, PWK reports the following disclosures for the last 24‐month period: he has investment interest in Convergent Therapeutics Inc, Cogent Biosciences, Context Therapeutics LLC, DRGT, Mirati, Placon, PrognomIQ, Seer Biosciences, SnyDevRx, and XLink, he is a company board member for Convergent Therapeutics Inc., Context Therapeutics LLC, he is a company founder for Convergent Therapeutics Inc and XLink and is/was a consultant/scientific advisory board member for Anji, Bavarian Nordic Immunotherapeutics, Candel, DRGT, Immunis, AI (previously OncoCellMDX), Janssen, Progenity, PrognomIQ, Seer Biosciences, SynDevRX, Tarveda Therapeutics, Veru, and serves on data safety monitoring boards for Genentech/Roche and Merck. He reports spousal association with Bayer.

## Author contributions

YZM and PWK conceived and designed the study. YZM and YL involved in development of methodology. YZM, YL, LEJ, SN, TAG, RG, BG, G‐SML, J‐HL, SRC, LAM, and FYF contributed to acquisition of data (acquired and managed patient data, provided facilities, etc.). YZM, YL, SN, and MS contributed to analysis and interpretation of data (e.g., statistical analysis, biostatistics, computational analysis). YZM and PWK contributed to writing, review, and/or revision of the manuscript. YZM, YL, SN, and PWK contributed to administrative, technical, or material support (i.e., reporting or organizing data, constructing databases). YZM and PWK supervised the study.

### Peer Review

The peer review history for this article is available at https://publons.com/publon/10.1002/1878‐0261.13140.

## Supporting information


**Fig. S1.** Genomic alterations of *SNAI2, NCOA2,* and *MYC* are correlated with poor clinical outcomes in PC.

**Fig. S2.** Amplification of *SNAI2* is correlated with poor clinical outcomes in PC.

**Fig. S3.** Correlation between *SNAI2* levels, copy number alterations, and fraction genome alterations in PC.

**Fig. S4.** *TMPRSS2‐ERG* is involved in the epigenetic silencing of its targets in PC.

**Fig. S5.** Correlation between *SNAI2* levels and different clinical attributes in the TCGA and SU2C cohorts.

**Fig. S6.** Silencing of SNAI2 is required for tumor cell proliferation.

**Fig. S7.** Silencing of SNAI2 contributes to luminal differentiation in PC.

**Fig. S8.** *SNAI2* interacts with the tumor microenvironment in PC.

**Fig. S9.** *SNAI2* levels determine dasatinib sensitivity.

**Table S1.** Summary of 18 prostate cancer clinical cohorts.

**Table S2.** The reagents and cell lines used in the study.

**Table S3.** The correlation of *SNAI2* expression levels with overall survival in TCGA cancer cohorts.

**Table S4.** The correlations between *SNAI2* mRNA with its methylation and ERG mRNA in TCGA cancer cohorts.

**Table S5.** Enriched gene sets (Mg signature: C2) in low and high *SNAI2* groups in TCGA and SU2C cohorts.
