# Supplementary material for: Dynamic expression of SNAI2 in prostate cancer predicts tumor progression and drug sensitivity
Source: Mol Oncol. 2022 Feb 11;16(13):2451–69. doi: 10.1002/1878-0261.13140 (PMC9251866; doi:10.1002/1878-0261.13140)
Supplement: Supplementary file 1 — Fig. S1. Genomic alterations of SNAI2, NCOA2, and MYC are correlated with poor clinical outcomes in PC. [file MOL2-16-2451-s008.pdf]

Fig. S1

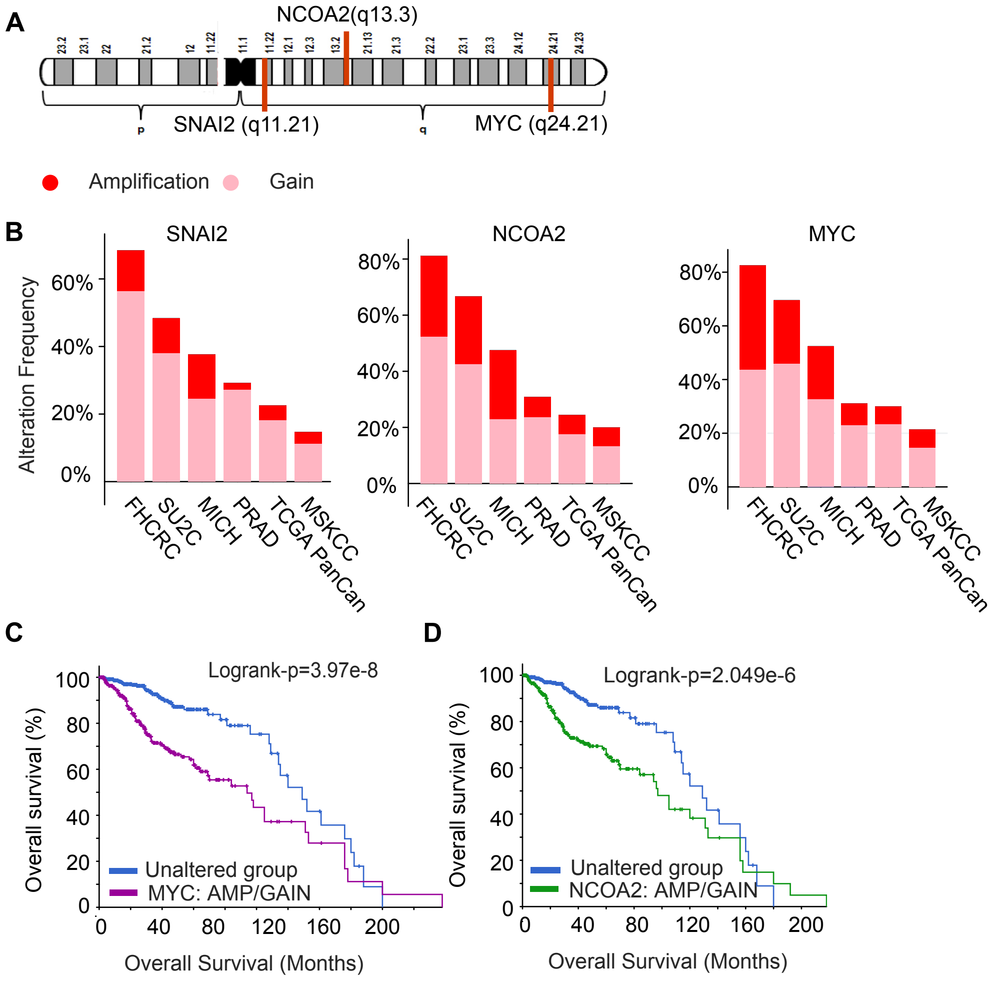

Figure S1. Genomic alterations of SNAI2, NCOA2, and MYC are correlated with poor clinical outcomes in PC. A, The genomic location of SNAI2, NCOA2, and MYC on chromosome 8. B, The genomic alteration frequency of SNAI2, NCOA2, and MYC in 6 PC cohorts. The data were extracted from cBioPortal. C-D, Correlation between NCOA2 and MYC amplification, respectively, and overall survival in the 6 PC cohorts.
