# Supplementary material for: Dynamic expression of SNAI2 in prostate cancer predicts tumor progression and drug sensitivity
Source: Mol Oncol. 2022 Feb 11;16(13):2451–69. doi: 10.1002/1878-0261.13140 (PMC9251866; doi:10.1002/1878-0261.13140)
Supplement: Supplementary file 2 — Fig. S2. Amplification of SNAI2 is correlated with poor clinical outcomes in PC. [file MOL2-16-2451-s010.pdf]

Figure S2

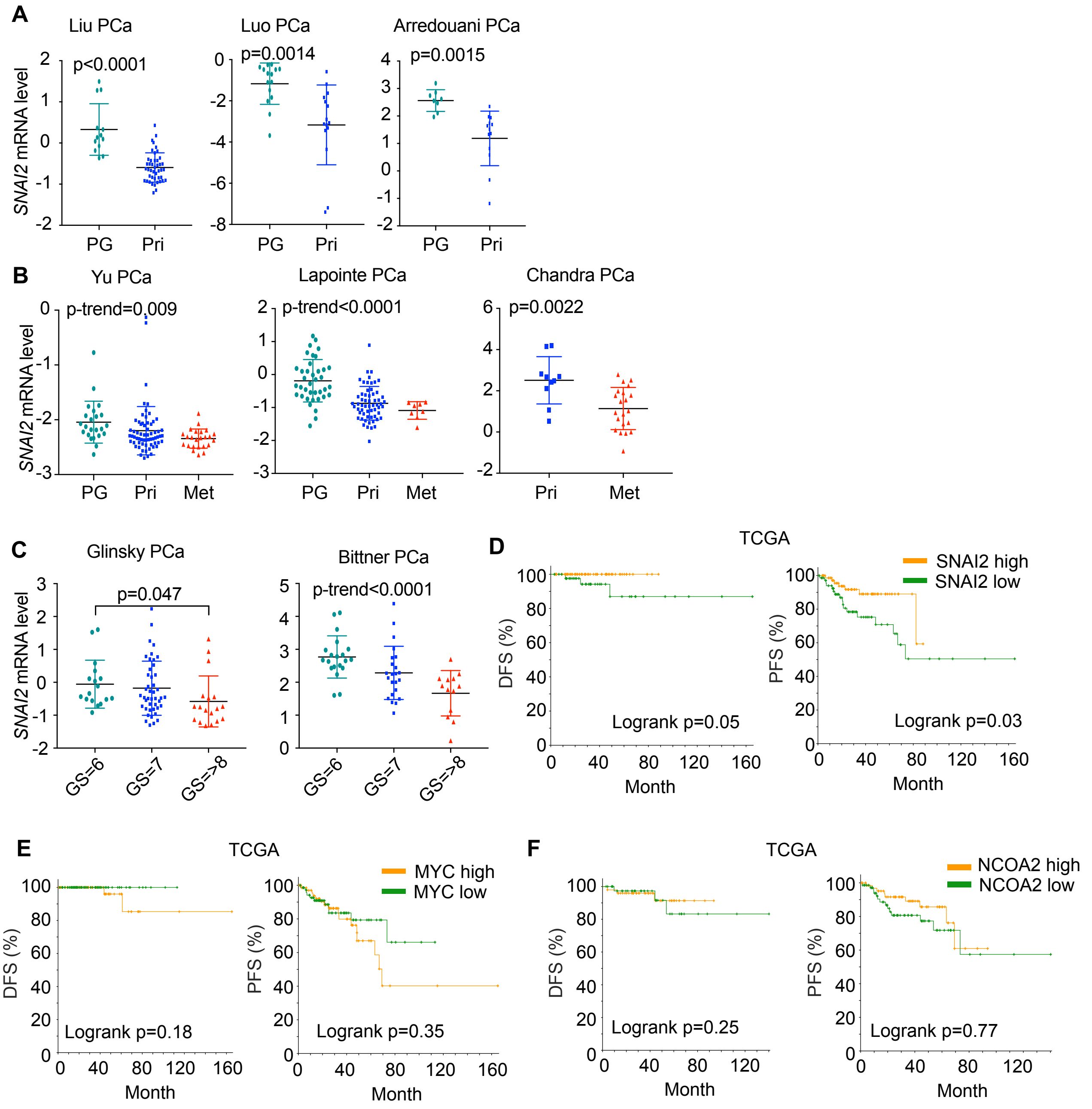

Figure S2. Amplification of SNAI2 is correlated with poor clinical outcomes in PC. A and B, Correlation between SNAI2 levels and tumor progression in 6 PC cohorts. The data were extracted using Oncomine. C, Correlation between SNAI2 levels and Gleason grades in 2 PC cohorts. D-F, The correlation of SNAI2 (D), MYC (E), and NCOA2 (F) protein expression with disease-free and progression-free survival in TCGA cohort.
