# Supplementary material for: Dynamic expression of SNAI2 in prostate cancer predicts tumor progression and drug sensitivity
Source: Mol Oncol. 2022 Feb 11;16(13):2451–69. doi: 10.1002/1878-0261.13140 (PMC9251866; doi:10.1002/1878-0261.13140)
Supplement: Supplementary file 3 — Fig. S3. Correlation between SNAI2 levels, copy number alterations, and fraction genome alterations in PC. [file MOL2-16-2451-s007.pdf]

Fig. S3

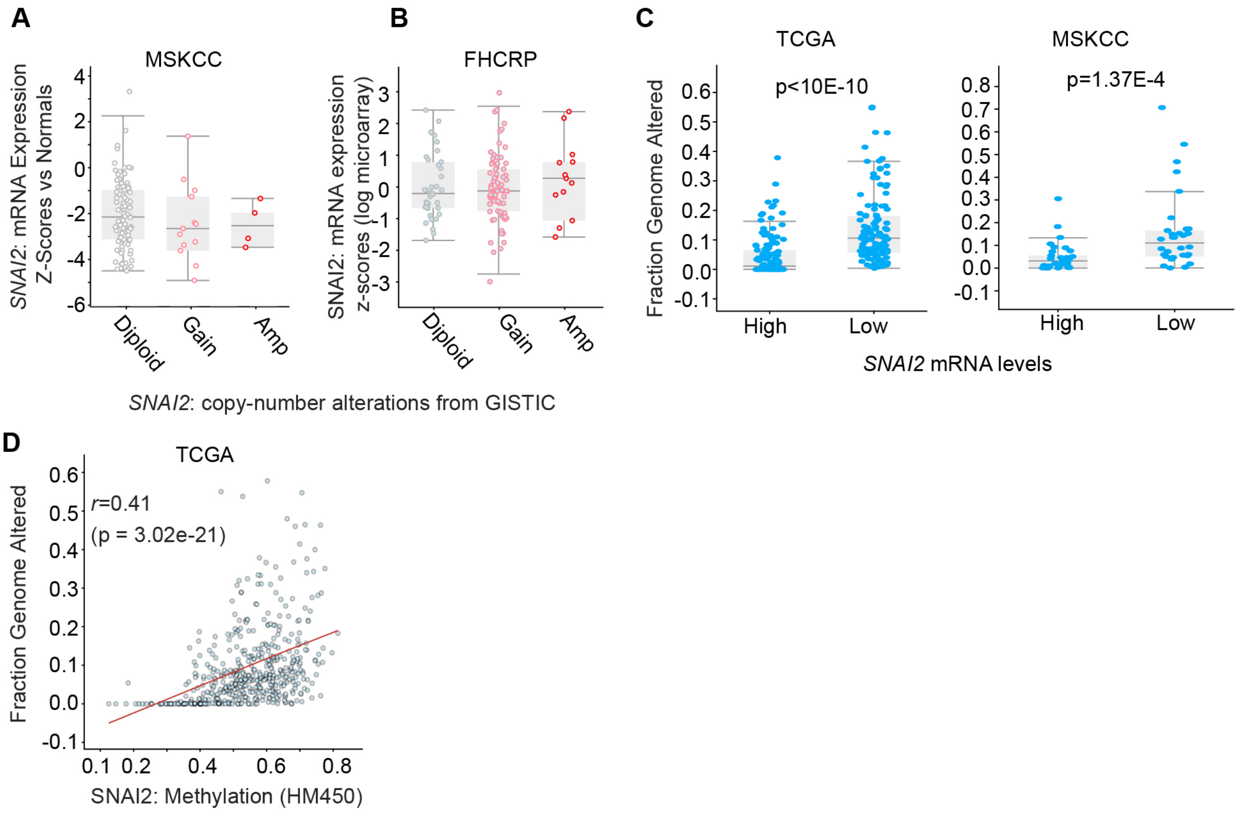

Figure S3. Correlation between SNAI2 levels, copy number alterations, and fraction genome alterations in PC. A and B, Correlation between copy number alterations and mRNA levels of SNAI2 in the MSKCC and FHCRP cohorts. C, Correlation between fraction genome alteration signal and SNAI2 levels in the TCGA and MSKCC cohorts. D, Correlation between fraction genome alteration signal and methylation of SNAI2 in the TCGA cohort.
