# Supplementary material for: Dynamic expression of SNAI2 in prostate cancer predicts tumor progression and drug sensitivity
Source: Mol Oncol. 2022 Feb 11;16(13):2451–69. doi: 10.1002/1878-0261.13140 (PMC9251866; doi:10.1002/1878-0261.13140)
Supplement: Supplementary file 4 — Fig. S4. TMPRSS2‐ERG is involved in the epigenetic silencing of its targets in PC. [file MOL2-16-2451-s001.pdf]

Fig. S4

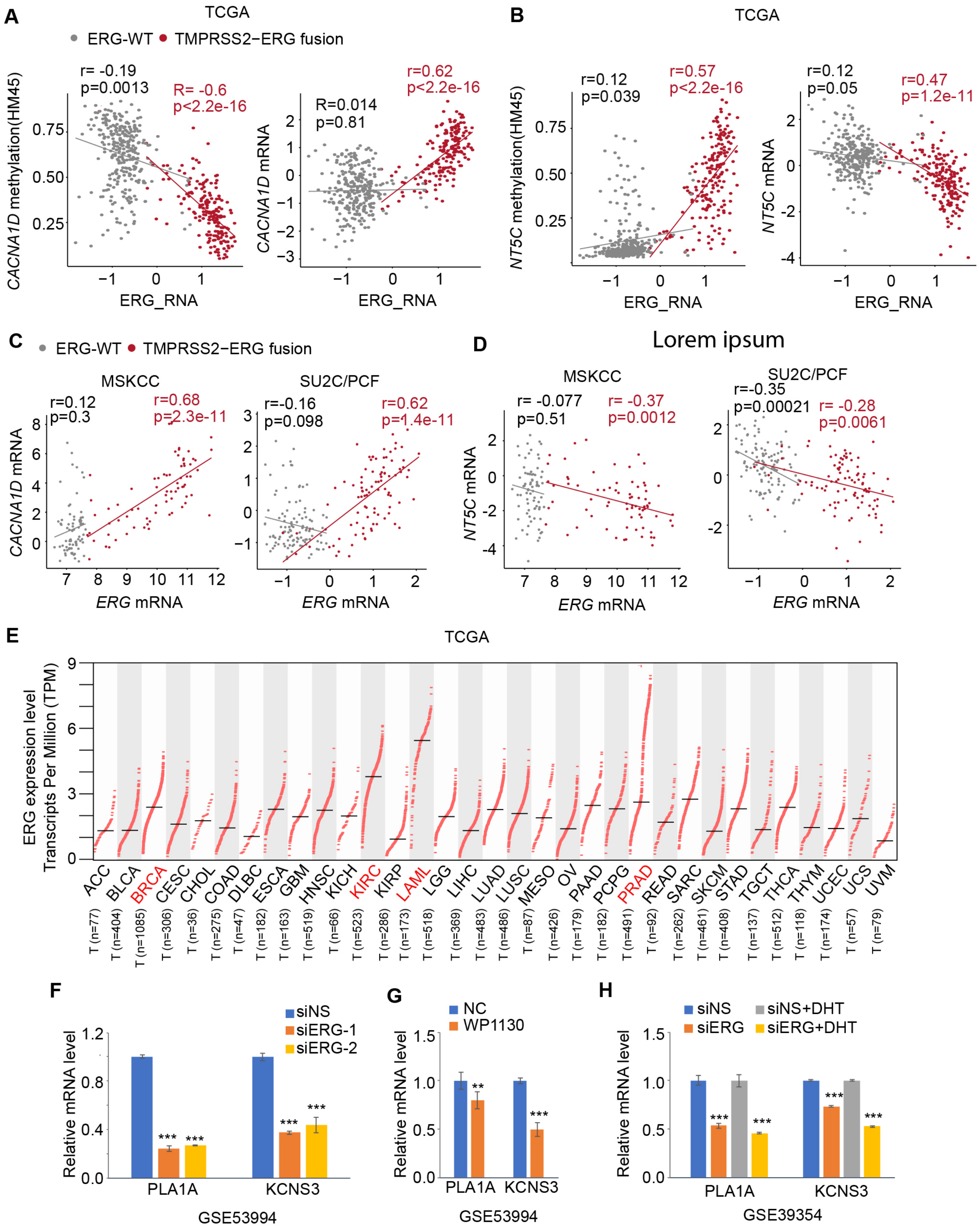
A
TCGA
ERG-WT TMPRSS2−ERG fusion
r= -0.19
p=0.0013
R= -0.6
p<2.2e-16
CACNA1D methylation(HM45)
ERG_RNA
r=0.62
p<2.2e-16
R=0.014
p=0.81
CACNA1D mRNA
ERG_RNA
B
TCGA
r=0.12
p=0.039
r=0.57
p<2.2e-16
NT5C methylation(HM45)
ERG_RNA
r=0.12
p=0.05
r=0.47
p=1.2e-11
NT5C mRNA
ERG_RNA
C
ERG-WT TMPRSS2−ERG fusion
MSKCC
r=0.12
p=0.3
r=0.68
p=2.3e-11
CACNA1D mRNA
ERG mRNA
SU2C/PCF
r=-0.16
p=0.098
r=0.62
p=1.4e-11
ERG mRNA
D
Lorem ipsum
MSKCC
r= -0.077
p=0.51
r= -0.37
p=0.0012
NT5C mRNA
ERG mRNA
SU2C/PCF
r=-0.35
p=0.00021
r= -0.28
p=0.0061
ERG mRNA
E
TCGA
ERG expression level
Transcripts Per Million (TPM)
ACC BLCA BRCA CESC CHOL COAD DLBC ESCA GBM HNSC KICH KIRC KIRP LAML LGG LIHC LUAD LUSC MESO OV PAAD PCPG PRAD READ SARC SKCM STAD TGCT THCA THYM UCEC UCS UVM
T (n=77) T (n=404) T (n=1085) T (n=306) T (n=36) T (n=275) T (n=47) T (n=182) T (n=163) T (n=519) T (n=66) T (n=523) T (n=286) T (n=173) T (n=518) T (n=369) T (n=483) T (n=486) T (n=87) T (n=426) T (n=179) T (n=182) T (n=491) T (n=92) T (n=262) T (n=461) T (n=408) T (n=137) T (n=512) T (n=118) T (n=174) T (n=57) T (n=79)
F
siNS
siERG-1
siERG-2
Relative mRNA level
PLA1A KCNS3
GSE53994
G
NC
WP1130
Relative mRNA level
PLA1A KCNS3
GSE53994
H
siNS
siERG
siNS+DHT
siERG+DHT
Relative mRNA level
PLA1A KCNS3
GSE39354

Figure S4. TMPRSS2-ERG is involved in the epigenetic silencing of its targets in PC. A and B, Correlation between ERG levels and methylation and mRNA levels, respectively, of CACAN1D (A) and NT5C (B) levels in the TCGA cohort. C and D, Correlation between ERG levels and mRNA levels of ERG targets (CACAN1D (C) and NT5C (D)) in the MSKCC and SU2C/PCF cohorts. E, ERG gene expression across multiple cancer types. The data were extracted from GEPIA (http://gepia.cancer-pku.cn/). F-H, The effects on ERG targets (PLA1A and KCNS3) expression by inhibition of ERG by siRNAs or WP130 in VCaP cells. The datasets from GSE53994 (F, G) and GSE39354 (H) were used for the analysis. Figure values represent the mean ± SE of duplicates. **, $P < 0.01$; ***, $P < 0.001$; vs. control groups treated with nonspecific (siNS) siRNA or DMSO.
