# Supplementary material for: Dynamic expression of SNAI2 in prostate cancer predicts tumor progression and drug sensitivity
Source: Mol Oncol. 2022 Feb 11;16(13):2451–69. doi: 10.1002/1878-0261.13140 (PMC9251866; doi:10.1002/1878-0261.13140)
Supplement: Supplementary file 5 — Fig. S5. Correlation between SNAI2 levels and different clinical attributes in the TCGA and SU2C cohorts. [file MOL2-16-2451-s004.pdf]

Figure S5

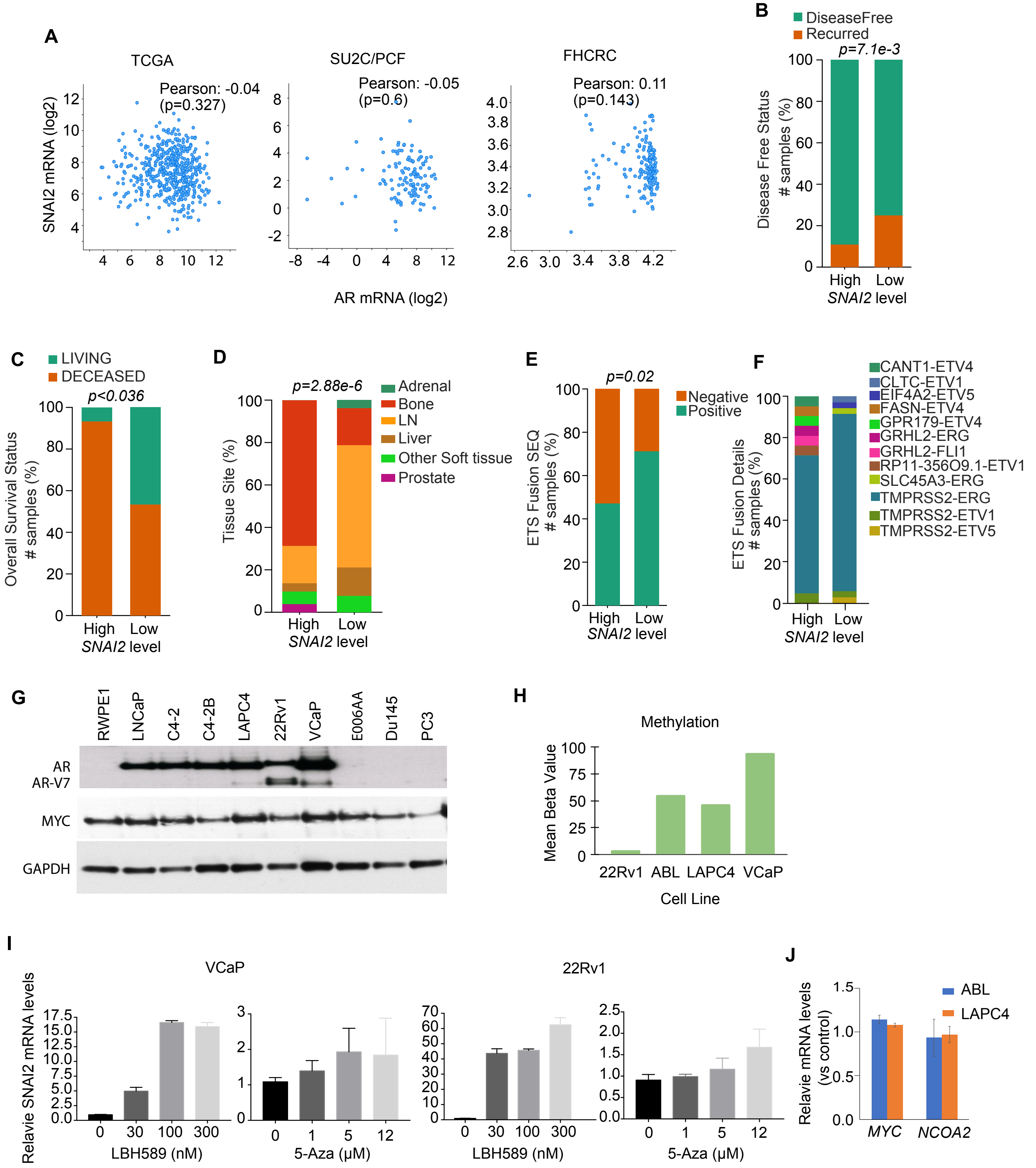

A
TCGA
SU2C/PCF
FHCRC
Pearson: -0.04 (p=0.327)
Pearson: -0.05 (p=0.6)
Pearson: 0.11 (p=0.143)
SNAI2 mRNA (log2)
AR mRNA (log2)
B
DiseaseFree
Recurred
p=7.1e-3
Disease Free Status # samples (%)
High Low
SNAI2 level
C
LIVING
DECEASED
p<0.036
Overall Survival Status # samples (%)
D
p=2.88e-6
Adrenal
Bone
LN
Liver
Other Soft tissue
Prostate
Tissue Site (%)
E
p=0.02
Negative
Positive
ETS Fusion SEQ # samples (%)
F
CANT1-ETV4
CLTC-ETV1
EIF4A2-ETV5
FASN-ETV4
GPR179-ETV4
GRHL2-ERG
GRHL2-FLI1
RP11-356O9.1-ETV1
SLC45A3-ERG
TMPRSS2-ERG
TMPRSS2-ETV1
TMPRSS2-ETV5
ETS Fusion Details # samples (%)
G
RWPE1
LNCaP
C4-2
C4-2B
LAPC4
22Rv1
VCaP
E006AA
Du145
PC3
AR
AR-V7
MYC
GAPDH
H
Methylation
Mean Beta Value
22Rv1
ABL
LAPC4
VCaP
Cell Line
I
VCaP
22Rv1
Relavie SNAI2 mRNA levels
LBH589 (nM)
5-Aza (μM)
J
ABL
LAPC4
Relavie mRNA levels (vs control)
MYC
NCOA2

Figure S5. Correlation between SNAI2 levels and different clinical attributes in the TCGA and SU2C cohorts. A, Correlation between SNAI2 and AR levels in primary and metastatic PC tumors. B, Correlation between SNAI2 levels and disease-free status in TCGA. C and D, Correlation between SNAI2 levels and lethality (C) and metastatic sites (D) in the SU2C cohort. E and F, Correlation between ETS fusion status and SNAI2 levels in SU2C (E). The ETS fusion type details were shown in F. G, AR, AR-V7 protein, and MYC expression in PC cell lines. H, Methylation status of SNAI2 in PC cell lines. The methylation status was detected by genomic methylation-seq. I, Effects of DNMTi (5-Aza) and HDACi (LBH589) treatment on SNAI2 levels in VCaP and 22Rv1 cells. The SNAI2 mRNA levels were detected after 5 days of treatment with 5-Aza or 1 day of treatment with LBH589. J, Effects of LBH589 treatment on MYC and NCOA2 mRNA levels in ABL and LAPC4 cells. The mRNA levels were detected after 1 day of treatment with LBH589n (300nM). Relative mRNA levels were normalized with DMSO control groups. Figure values represent the mean ± SE of three independent experiments. **, $P < 0.01$; ***, $P < 0.001$; vs. control groups treated with DMSO.
