# Supplementary material for: Dynamic expression of SNAI2 in prostate cancer predicts tumor progression and drug sensitivity
Source: Mol Oncol. 2022 Feb 11;16(13):2451–69. doi: 10.1002/1878-0261.13140 (PMC9251866; doi:10.1002/1878-0261.13140)
Supplement: Supplementary file 6 — Fig. S6. Silencing of SNAI2 is required for tumor cell proliferation. [file MOL2-16-2451-s013.pdf]

Fig. S6

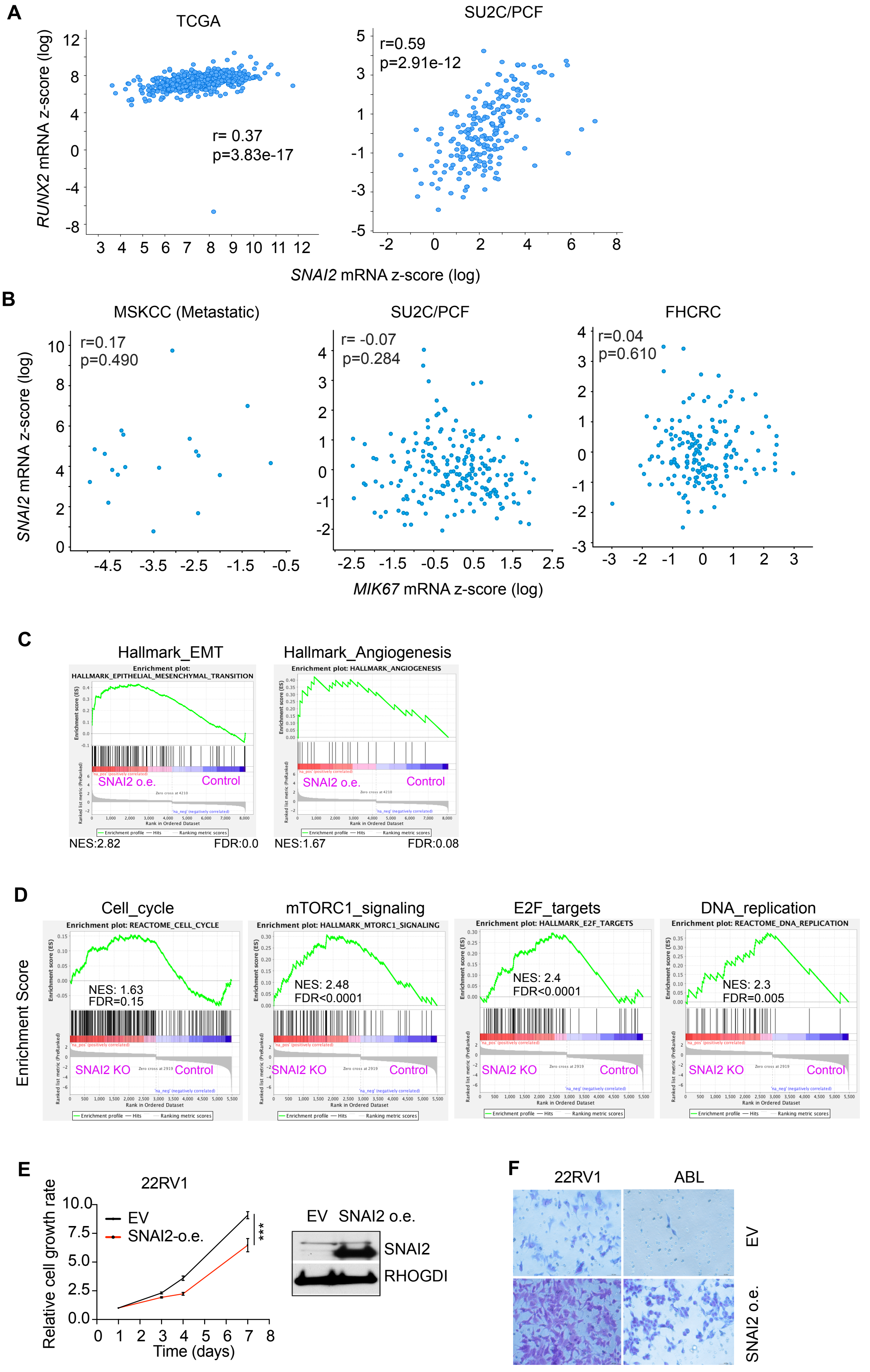
A
TCGA
SU2C/PCF
RUNX2 mRNA z-score (log)
SNAI2 mRNA z-score (log)
r= 0.37
p=3.83e-17
r=0.59
p=2.91e-12
B
MSKCC (Metastatic)
SU2C/PCF
FHCRC
SNAI2 mRNA z-score (log)
MIK67 mRNA z-score (log)
r=0.17
p=0.490
r= -0.07
p=0.284
r=0.04
p=0.610
C
Hallmark_EMT
Hallmark_Angiogenesis
Enrichment plot: HALLMARK_EPITHELIAL_MESENCHYMAL_TRANSITION
Enrichment plot: HALLMARK_ANGIOGENESIS
SNAI2 o.e.
Control
NES:2.82
FDR:0.0
NES:1.67
FDR:0.08
D
Enrichment Score
Cell_cycle
mTORC1_signaling
E2F_targets
DNA_replication
Enrichment plot: REACTOME_CELL_CYCLE
Enrichment plot: HALLMARK_MTORC1_SIGNALING
Enrichment plot: HALLMARK_E2F_TARGETS
Enrichment plot: REACTOME_DNA_REPLICATION
NES: 1.63
FDR=0.15
NES: 2.48
FDR<0.0001
NES: 2.4
FDR<0.0001
NES: 2.3
FDR=0.005
SNAI2 KO
Control
E
22RV1
Relative cell growth rate
Time (days)
EV
SNAI2-o.e.
***
EV
SNAI2 o.e.
SNAI2
RHOGDI
F
22RV1
ABL
EV
SNAI2 o.e.

Figure S6. Silencing of SNAI2 is required for tumor cell proliferation. A, Correlation between RUNX2 levels and SNAI2 gene expression in primary and metastatic PC cohorts. B, Correlation between SNAI2 levels and MKI67 expression in metastatic PC cohorts. B and C, Metastatic pathways (B) and cell proliferation related pathways (C) are enriched in cells overexpressing SNAI2. The dataset was extracted from GSE80042. D, The effects on cell proliferation of 22Rv1 cells overexpressing SNAI2. Cell viability was detected in 7 days. SNAI2 expression was detected by immunoblotting. E, Invasion assay of 22Rv1 and ABL cells overexpressing SNAI2. Figure values represent the mean ± SE of three independent experiments. ***, $P < 0.001$; vs. control groups infected with empty vector (EV).
