# Supplementary material for: Dynamic expression of SNAI2 in prostate cancer predicts tumor progression and drug sensitivity
Source: Mol Oncol. 2022 Feb 11;16(13):2451–69. doi: 10.1002/1878-0261.13140 (PMC9251866; doi:10.1002/1878-0261.13140)
Supplement: Supplementary file 7 — Fig. S7. Silencing of SNAI2 contributes to luminal differentiation in PC. [file MOL2-16-2451-s002.pdf]

Fig. S7

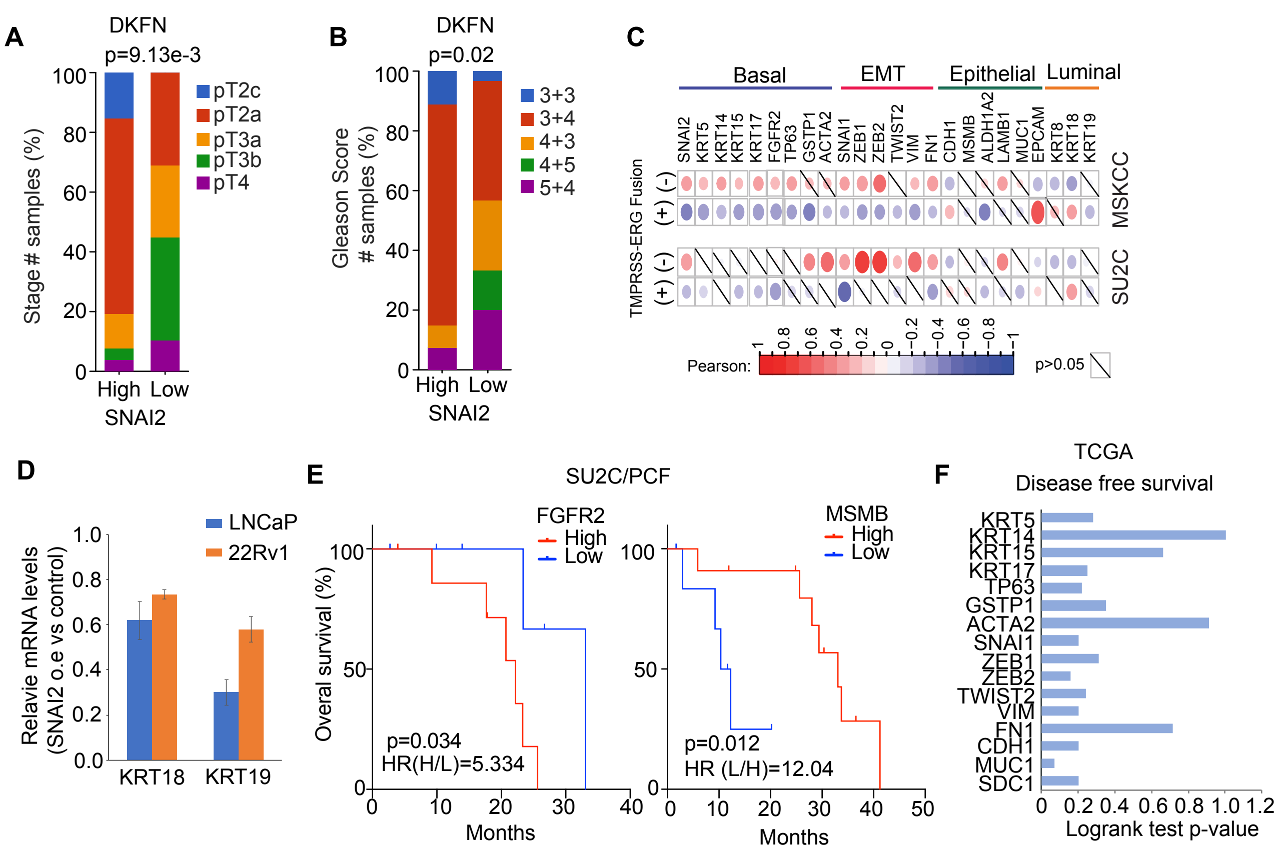

Figure S7. Silencing of SNAI2 contributes to luminal differentiation in PC. A and B, Correlation between SNAI2 levels and disease stages (A) and Gleason grades (B) in the DKFN cohort. C, Correlation between ERG expression and mRNA levels of lineage markers in the MSKCC and SU2C cohorts. D, the regulation of luminal markers (KRT18, KRT19) by SNAI2 overexpression in LNCaP and 22Rv1 cells. The relative mRNA levels were normalized to empty vector group (control). E and F, Correlation between lineage markers and overall survival in SU2C/PCF (E) and disease-free survival in TCGA (F).
