# Supplementary material for: Dynamic expression of SNAI2 in prostate cancer predicts tumor progression and drug sensitivity
Source: Mol Oncol. 2022 Feb 11;16(13):2451–69. doi: 10.1002/1878-0261.13140 (PMC9251866; doi:10.1002/1878-0261.13140)
Supplement: Supplementary file 8 — Fig. S8. SNAI2 interacts with the tumor microenvironment in PC. [file MOL2-16-2451-s014.pdf]

Fig. S8

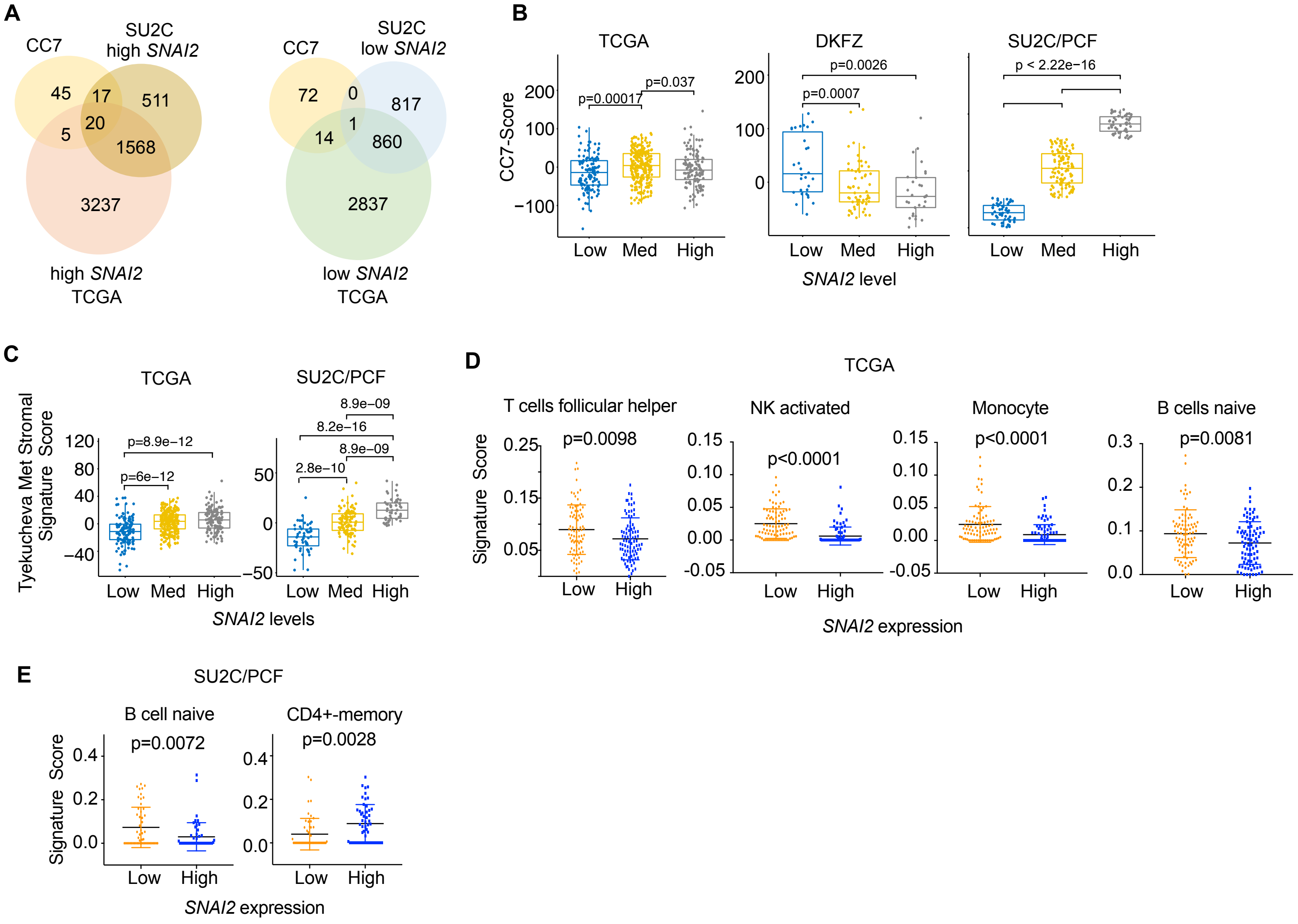

Figure S8. SNAI2 interacts with the tumor microenvironment in PC. A, Common genes between the CC7 signature and enriched genes in high- or low-SNAI2 groups from the TCGA and SU2C cohorts. B, Correlation between SNAI2 levels and CC7 signature scores in the TCGA, DKFN, and SU2C/PCF cohorts. C, Correlation between SNAI2 levels and Tyekucheva metastasis stromal signature score in the TCGA and SU2C cohorts. D and E, Profiling of immune cells by CIBERSORT in the TCGA (D) and SU2C/PCF (E) cohorts. Significance was determined using Wilcoxon's rank‐sum test.
