# Supplementary material for: Dynamic expression of SNAI2 in prostate cancer predicts tumor progression and drug sensitivity
Source: Mol Oncol. 2022 Feb 11;16(13):2451–69. doi: 10.1002/1878-0261.13140 (PMC9251866; doi:10.1002/1878-0261.13140)
Supplement: Supplementary file 9 — Fig. S9. SNAI2 levels determine dasatinib sensitivity. [file MOL2-16-2451-s011.pdf]

Fig. S9

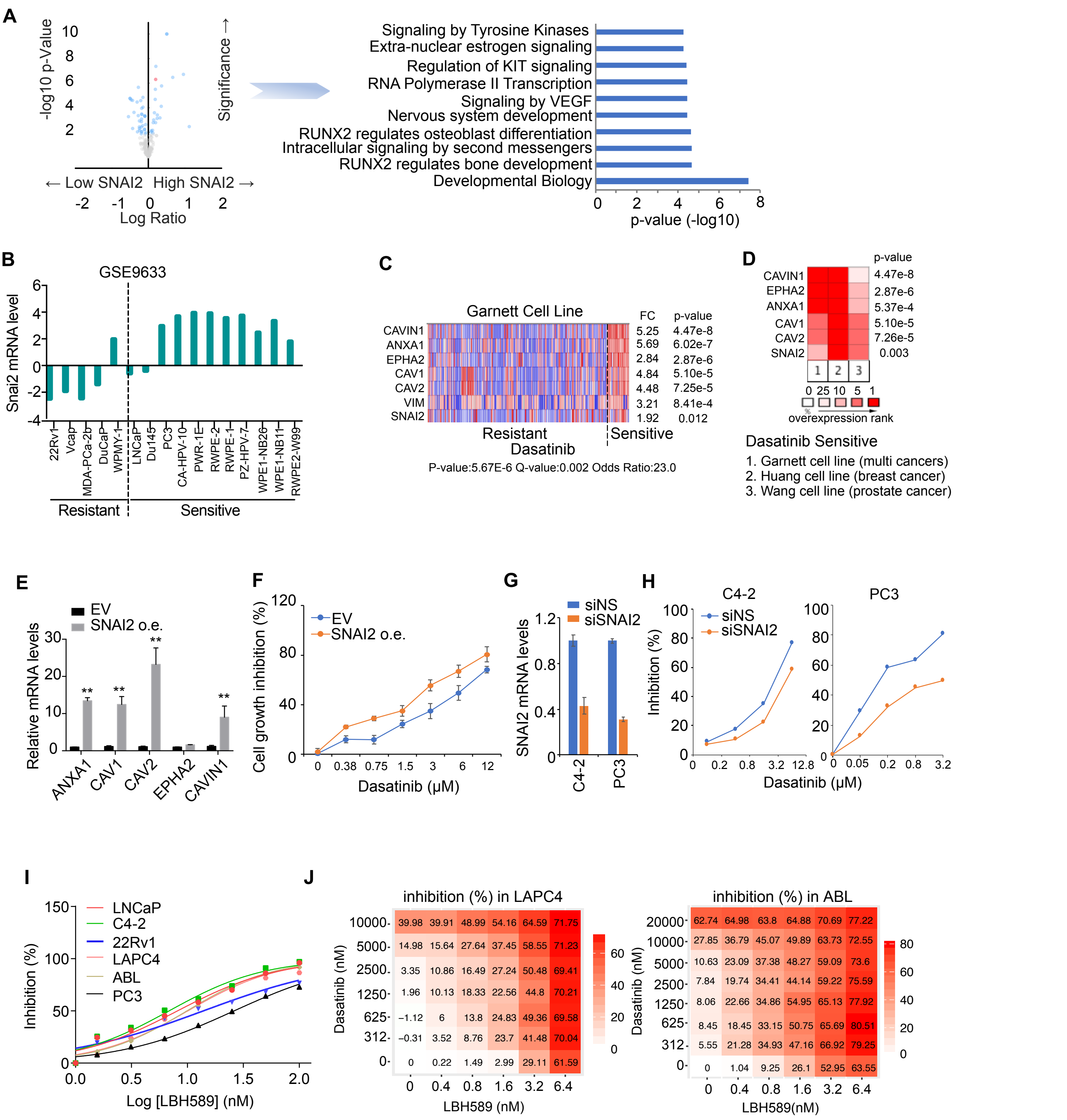

Figure S9. SNAI2 levels determine dasatinib sensitivity. A, Pathway enrichment of proteins that are positively correlated with high SNAI2 expression in TCGA. The data were extracted from cBioPortal. B, Dasatinib sensitivity of normal prostate and tumor cell lines. The data was extracted from GSE9633. C, The 5-gene signature plus SNAI2 profiling in Garnett cell line dataset (of multiple cancers). D, Overexpression of the 5-gene signature and SNAI2 in dasatinib-sensitive cells from 3 cell line datasets (multiple cancers, breast cancer, and PC). E, Regulation of a 5-gene signature by 22Rv1 cells overexpressing SNAI2. Gene expression was detected by qRT-PCR. F, Overexpression of SNAI2 in LNCaP cells increased the sensitivity to dasatinib. Cell growth inhibition was detected after 3 days of treatment with dasatinib. G, SNAI2 mRNA expression in C4-2 and PC3 cells with siNS (non-specific) and siSNAI2 transfection. H, Cell growth inhibition was induced by dasatinib in C4-2 and PC3 cells with siNS and siSNAI2 transfection. The inhibition efficacy was calculated against DMSO groups in siNS or siSNAI2 treatment after 3 days of treatment with dasatinib. I, LBH589 sensitivity in 6 PC cell lines. Cell viability was detected after LBH589 treatment for 1 day. J, The drug response matrix of combination of dasatinib and LBH589 in LAPC4 and ABL cells. Figure values represent the mean ± SE of three independent experiments. *, P <0.05; **, P < 0.01; vs. control groups infected with empty vector (EV).
