# Supplementary material for: Dynamic expression of SNAI2 in prostate cancer predicts tumor progression and drug sensitivity
Source: Mol Oncol. 2022 Feb 11;16(13):2451–69. doi: 10.1002/1878-0261.13140 (PMC9251866; doi:10.1002/1878-0261.13140)
Supplement: Supplementary file 10 — Table S1. Summary of 18 prostate cancer clinical cohorts. [file MOL2-16-2451-s012.docx]

| **Table S1. Summary of 18 prostate cancer clinical cohorts** | | | | | | | | | |
| --- | --- | --- | --- | --- | --- | --- | --- | --- | --- |
| **Cohort name** | **Numbers of types of samples in cohort suitable for use in this analysis** | | | | **Clinical outcome** | **Data type** | **Year** | **Journal** | **Reference** |
|  | **Benign/ normal tissue** | **Tumor** | **primary** | **meta-static** |  |  |  |  |  |
| TCGA (Pan Cancer) | 0 | 494 | 494 | 0 | OS, BCR, PFS | WES, RNA-seq, RPPA | 2018 | Cell | S1 |
| PRAD (MSKCC/DFCI) | 0 | 1013 | 680 | 333 | N/A | WES | 2018 | Nat Genet | S2 |
| MSKCC | 29 normal | 218 | 181 | 37 | BCR | aCGH, RNA-seq | 2010 | Cancer Cell | S3 |
| SU2C/PCF | 0 | 444 | 0 | 444 | OS | WES, RNA-seq | 2019 | PNAS | S4 |
| FHCRC | 176 benign | 176 | 22 | 154 | BCR | aCGH, WES, microarray | 2016 | Nat Med | S5 |
| MICH (Grasso) | 28 benign | 94 | 59 | 35 | OS | aCGH, microarray | 2012 | Nature | S6 |
| Broad/Cornell | 0 | 57 | 55 | 2 | N/A | WGS | 2013 | Cell | S7 |
| Tomlins, et al. | 18 | 52 | 32 | 20 | N/A | microarray | 2007 | Nat Genet | S8 |
| Chandran, et al. | 0 | 31 | 10 | 21 | N/A | microarray | 2007 | BMC Cancer | S9 |
| Lapointe prostate | 41 normal | 71 | 62 | 9 | Biochemical recurrence, survival | microarray | 2004 | PNAS | S10 |
| Yu, et al. | 23 normal | 89 | 64 | 25 | Survival status | microarray | 2004 | J Clin Oncol | S11 |
| Liu, et al. | 16 normal | 58 | 58 | 0 | N/A | aCGH | 2009 | Nat Med | S12 |
| Glinsky, et al. | 0 | 79 | 79 | 0 | BCR | microarray | 2004 | J Clin Invest. | S13 |
| Luo, et al. | 15 | 15 | 15 | 0 | N/A | microarray | 2002 | Mol Carcinog | S14 |
| Arredouani, et al. | 8 | 13 | 13 | 0 | N/A | microarray | 2009 | Clin Cancer Res | S15 |
| PHS/HPFS | 0 | 404 | 404 | 0 | Survival status | microarray | 2002 | Cancer cell | S16, S17 |
| Bittner, et al. | 0 | 60 | 60 | 0 | N/A | microarray | 2005 | not published | Oncomine |
| DKFZ | 0 | 324 | 324 | 0 | BCR | WGS, RNA-seq | 2018 | Cancer Cell | S18 |

Abbreviations: aCGH, array comparative genomic hybridization; OS, overall survival; BCR, biochemical recurrence; PFS, progression-free survival; RPPA, reverse phase protein array; SU2C/PCF, Stand Up to Cancer/Prostate Cancer Foundation; TCGA, The Cancer Genome Atlas; WES, whole-exome sequencing. WGS, whole-genome sequencing.

**Supplemental References:**

S1. Liu J, Lichtenberg T, Hoadley KA, Poisson LM, Lazar AJ, Cherniack AD, et al. An integrated TCGA pan-cancer clinical data resource to drive high-quality survival outcome analytics. Cell. 2018; 173: 400–416.e11.

S2. Armenia J, Wankowicz SAM, Liu D, Gao J, Kundra R, Reznik E, et al. The long tail of oncogenic drivers in prostate cancer. Nat Genet. 2018; 50: 645–651. Erratum in: Nat Genet. 2019; 51: 1194.

S3. Taylor BS, Schultz N, Hieronymus H, Gopalan A, Xiao Y, Carver BS, et al. Integrative genomic profiling of human prostate cancer. Cancer Cell. 2010; 18: 11–22.

S4. Abida W, Cyrta J, Heller G, Prandi D, Armenia J, Coleman I, et al. Genomic correlates of clinical outcome in advanced prostate cancer. Proc Natl Acad Sci U S A. 2019; 116: 11428–11436.

S5. Kumar A, Coleman I, Morrissey C, Zhang X, True LD, Gulati R, et al. Substantial interindividual and limited intraindividual genomic diversity among tumors from men with metastatic prostate cancer. Nat Med. 2016; 22: 369–378.

S6. Grasso CS, Wu YM, Robinson DR, Cao X, Dhanasekaran SM, Khan AP, et al. The mutational landscape of lethal castration-resistant prostate cancer. Nature. 2012; 487: 239–243.

S7. Baca SC, Prandi D, Lawrence MS, Mosquera JM, Romanel A, Drier Y, et al. Punctuated evolution of prostate cancer genomes. Cell. 2013; 153: 666–677.

S8: Tomlins SA, Mehra R, Rhodes DR, Cao X, Wang L, Dhanasekaran SM, et al. Integrative molecular concept modeling of prostate cancer progression. Nat Genet. 2007; 39: 41–51.

S9. Chandran UR, Ma C, Dhir R, Bisceglia M, Lyons-Weiler M, Liang W, et al. Gene expression profiles of prostate cancer reveal involvement of multiple molecular pathways in the metastatic process. BMC Cancer. 2007; 7: 64.

S10. Lapointe J, Li C, Higgins JP, van de Rijn M, Bair E, Montgomery K, et al. Gene expression profiling identifies clinically relevant subtypes of prostate cancer. Proc Natl Acad Sci U S A. 2004; 101: 811–816.

S11. Yu YP, Landsittel D, Jing L, Nelson J, Ren B, Liu L, et al. Gene expression alterations in prostate cancer predicting tumor aggression and preceding development of malignancy. J Clin Oncol. 2004; 22: 2790–2799.

S12. Liu W, Laitinen S, Khan S, Vihinen M, Kowalski J, Yu G, et al. Copy number analysis indicates monoclonal origin of lethal metastatic prostate cancer. Nat Med. 2009; 15: 559–565. Erratum in: Nat Med. 2009; 15: 819.

S13. Glinsky GV, Glinskii AB, Stephenson AJ, Hoffman RM, Gerald WL. Gene expression profiling predicts clinical outcome of prostate cancer. J Clin Invest. 2004; 113: 913–923.

S14. Luo JH, Yu YP, Cieply K, Lin F, Deflavia P, Dhir R, et al. Gene expression analysis of prostate cancers. Mol Carcinog. 2002; 33: 25–35.

S15. Arredouani MS, Lu B, Bhasin M, Eljanne M, Yue W, Mosquera JM, et al. Identification of the transcription factor single-minded homologue 2 as a potential biomarker and immunotherapy target in prostate cancer. Clin Cancer Res. 2009; 15: 5794–5802.

S16. Giovannucci E, Liu Y, Platz EA, Stampfer MJ, Willett WC. Risk factors for prostate cancer incidence and progression in the Health Professionals Follow-up Study. Int J Cancer 2007;121:1571-8.

S17. Steering Committee of the Physicians' Health Study Research Group. Final report on the aspirin component of the ongoing Physicians' Health Study. N Engl J Med. 1989 Jul 20;321(3):129-35.

S18. Gerhauser C, et al. Molecular Evolution of Early-Onset Prostate Cancer Identifies Molecular Risk Markers and Clinical Trajectories.

Cancer Cell. 2018. Dec 10;34(6):996-1011.
